# Integrated multiomic analysis and high‐throughput screening reveal potential gene targets and synergetic drug combinations for osteosarcoma therapy

**DOI:** 10.1002/mco2.317

**Published:** 2023-07-12

**Authors:** Wenchao Zhang, Lin Qi, Zhongyue Liu, Shasha He, Cheng‐zhi Wang, Ying Wu, Lianbin Han, Zhenxin Liu, Zheng Fu, Chao Tu, Zhihong Li

**Affiliations:** ^1^ Department of Orthopedics The Second Xiangya Hospital Central South University Changsha China; ^2^ Hunan Key Laboratory of Tumor Models and Individualized Medicine The Second Xiangya Hospital Changsha China; ^3^ Department of Oncology The Second Xiangya Hospital Central South University Changsha China; ^4^ MegaRobo Technologies Co., Ltd Suzhou China

**Keywords:** drug combination, high‐throughput screen, multiomic analysis, osteosarcoma, therapeutic target

## Abstract

Although great advances have been made over the past decades, therapeutics for osteosarcoma are quite limited. We performed long‐read RNA sequencing and tandem mass tag (TMT)‐based quantitative proteome on osteosarcoma and the adjacent normal tissues, next‐generation sequencing (NGS) on paired osteosarcoma samples before and after neoadjuvant chemotherapy (NACT), and high‐throughput drug combination screen on osteosarcoma cell lines. Single‐cell RNA sequencing data were analyzed to reveal the heterogeneity of potential therapeutic target genes. Additionally, we clarified the synergistic mechanisms of doxorubicin (DOX) and HDACs inhibitors for osteosarcoma treatment. Consequently, we identified 2535 osteosarcoma‐specific genes and several alternative splicing (AS) events with osteosarcoma specificity and/or patient heterogeneity. Hundreds of potential therapeutic targets were identified among them, which showed the core regulatory roles in osteosarcoma. We also identified 215 inhibitory drugs and 236 synergistic drug combinations for osteosarcoma treatment. More interestingly, the multiomic analysis pointed out the pivotal role of HDAC1 and TOP2A in osteosarcoma. HDAC inhibitors synergized with DOX to suppress osteosarcoma both in vitro and in vivo. Mechanistically, HDAC inhibitors synergized with DOX by downregulating SP1 to transcriptionally modulate TOP2A expression. This study provided a comprehensive view of molecular features, therapeutic targets, and synergistic drug combinations for osteosarcoma.

## Graphical abstract

Based on multiomic approaches including long‐read RNA sequencing, next‐generation RNA sequencing, single‐cell RNA sequencing (scRNA‐Seq), tandem mass tag (TMT)‐based quantitative proteomics, and high‐throughput drug screening, we provided an integral molecular landscape and effective target–drug interactions for osteosarcoma. Meanwhile, we identified hundreds of effective synergistic drug combinations for osteosarcoma. We also demonstrated a novel mechanism for drug synergy of doxorubicin and HDACs inhibitor, which greatly benefit the development of new clinical drugs and strategies for osteosarcoma.

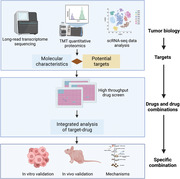

## 1 INTRODUCTION

Osteosarcoma is the most common primary bone cancer in children, adolescents, and young adults. It is a rare cancer type, with an annual incidence of 1−3 per million person‐years.^1^ It mostly occurs in the metaphysis of long bones near growth plates and less in the skull, jaw, or pelvis.^2^ The 5‐year event‐free survival for patients with localized osteosarcoma is approximately 70%, while for patients with metastatic or recurrent disease, it is less than 20%.^3^ The rarity of osteosarcoma greatly limits the development of new therapies targeting osteosarcoma.

The current standard neoadjuvant chemotherapy (NACT) for osteosarcoma treatment is mainly based on a three‐drug combination of methotrexate (MTX), doxorubicin (DOX; adriamycin), and cisplatin (DDP).^4^ However, the therapeutic effects of these drugs vary greatly among patients and accompanied with multiple side effects.^5^ Sorafenib, a multikinase inhibitor, has been added to the second‐line drugs for osteosarcoma.^6^ Clinical trials reported that 45% of patients with unresectable high‐grade osteosarcoma were progression free at 6 months when treated with the combination of sorafenib and everolimus.^7^ Further, drugs targeting core regulators or signaling pathways in osteosarcoma show therapeutic potential. For instance, the inhibitors of PI3K, mTOR, WEE1, and ATR yielded suppression of osteosarcoma cells.^1^, ^8^, ^9^ Immunotherapies, such as drugs targeting macrophages or improving immune infiltration, have also been investigated for osteosarcoma treatment. Monoclonal antibodies against the tumor membrane proteins RANKL and IGF‐1R have shown therapeutic potential in preclinical studies.^10^ Mifamurtide could activate innate immunity via the pattern‐recognition receptor NOD2, further providing therapeutic benefit for patients with recurrent and/or metastatic disease.^11^ However, immune checkpoint inhibitors, such as antibodies targeting PD‐1, showed poor responses in advanced osteosarcoma.^12^ Moreover, adoptive cell therapies (ACTs), such as chimeric antigen receptor T‐Cell (CAR‐T) therapy, have been introduced to osteosarcoma treatment. CAR‐T therapy targeting cell surface antigen disialoganglioside‐2 (GD2) could specifically recognize and kill osteosarcoma cells and is investigated in ongoing clinical trials.^13^ Other surface proteins such as CD166 and B7‐H3 have been reported as potential targets for CAR‐T therapy in preclinical studies.^14^, ^15^ Nevertheless, effective strategies for osteosarcoma therapy remain quite limited. A better understanding of the molecular characteristics of osteosarcoma is urgently required.

High‐throughput analyses provide an integral understanding of the molecular basis of tumors at multiomic levels in addition to phenotypic drug screening. For instance, whole exome sequencing demonstrated crucial genetic alterations in osteosarcoma.^16^ Clinical genomic sequencing of osteosarcoma using Integrated Mutation Profiling of Actionable Cancer Targets revealed distinct molecular subsets with potentially targetable alterations.^17^ A study showed highly heterogeneous somatic copy number alterations (SCNA) and structural rearrangements across osteosarcoma cases, suggesting the requirement of systematic genome information for SCNA‐based targeted therapy^18^ More recently, the single‐cell landscape of osteosarcoma has revealed intratumoral heterogeneity and an immunosuppressive microenvironment.^19^, ^20^ Previous proteomics studies in osteosarcoma mainly focused on cell lines or identified a quite limited number of proteins in clinical samples due to the limitation of proteomic approaches in the past.^21^, ^22^ High‐throughput drug screening previously identified several potential drugs for osteosarcoma.^23^ However, more studies of integral molecular and cellular analyses and drug synergy are urgently needed.

In this study, by utilizing multiomic approaches including long‐read RNA sequencing, next‐generation RNA sequencing, single‐cell RNA sequencing (scRNA‐Seq), and tandem mass tag (TMT)‐based quantitative proteomics in combination with high‐throughput drug screening, we provided an integral molecular landscape of osteosarcoma, with potential therapeutic drug targets and hundreds of effective synergistic drug combinations. Moreover, we revealed the molecular mechanism that HDAC inhibitors synergize with DOX by downregulating the transcription factor *SP1*, which further modulates *TOP2A* expression. These findings deepen our understanding of osteosarcoma biology and potential therapeutic targets, which could translate into clinical practice.

## 2 RESULT

### 2.1 Transcriptomic profiling of osteosarcoma

**FIGURE 1:**
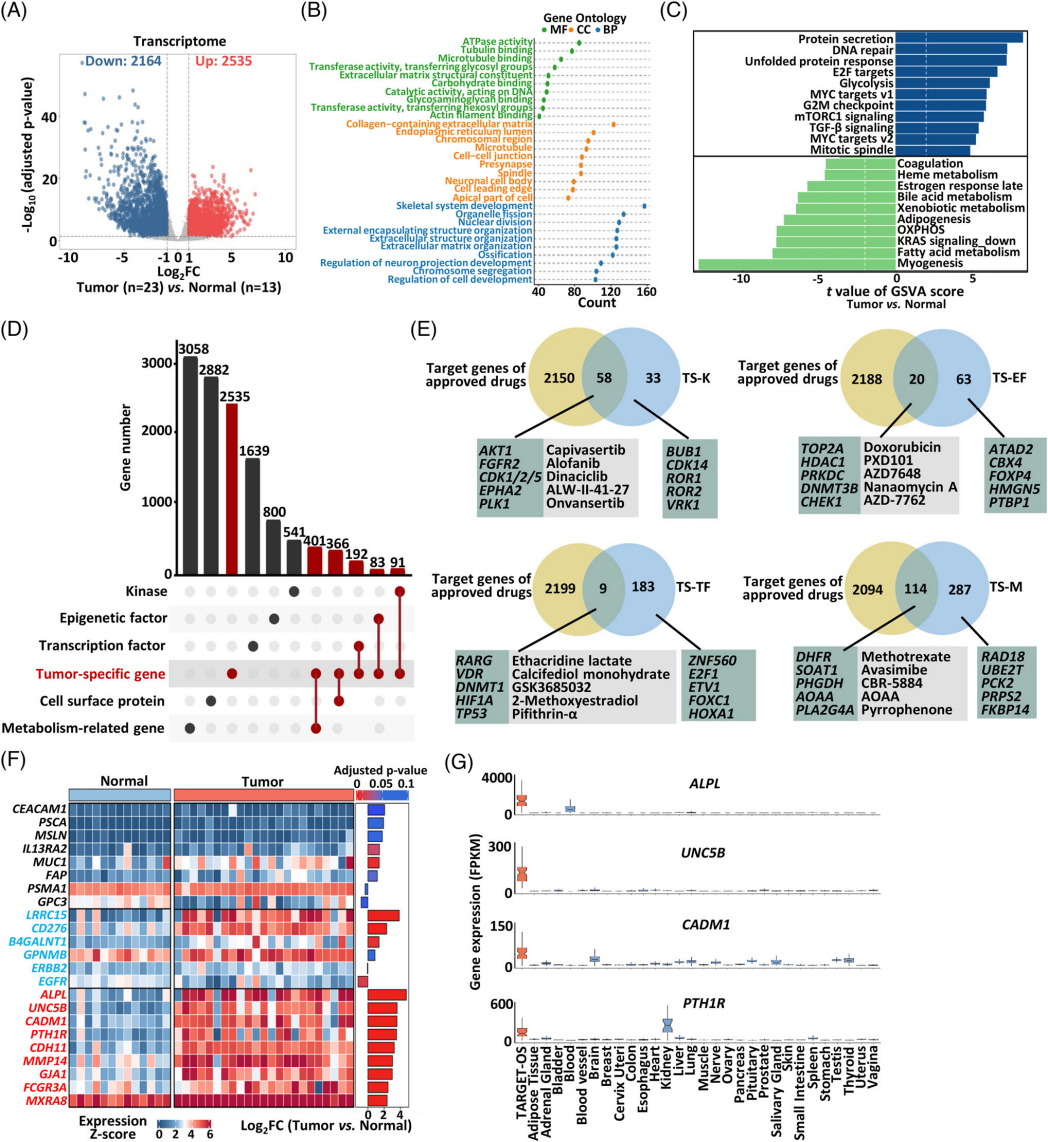
Transcriptomic profiling and potential therapeutic targets of osteosarcoma. (A) Volcano plot indicating significantly differentially expressed genes (DEGs) between osteosarcoma (*n* = 23) and the adjacent normal tissues (*n* = 13) (criteria: |fold change| ≥ 2, adjusted *p* value < 0.05). (B) Gene ontology (GO) analysis of the 2,535 DEGs upregulated in osteosarcoma. (C) Differences in hallmark pathways scored by gene set variation analysis (GSVA) between osteosarcoma (*n* = 23) and normal tissues (*n* = 13). (D) Number of tumor‐specific genes (TSGs) and a series of genes according to their biological functions and cellular locations. (E) Venn diagrams of overlapping genes between tumor‐specific annotated gene sets and the target genes of the approved drugs in the DrugBank. Examples of corresponding drugs were listed in the grey box. (F) Heatmap of mRNA expression of cell‐surface targets for adoptive cell therapy (ACT) between osteosarcoma (*n* = 23) and the normal adjacent tissues (*n* = 13). Black, commonly used targets for solid tumors. Blue, identified cell surface targets already investigated in the ACT of osteosarcoma. Red, novel cell surface genes specifically and highly expressed in osteosarcoma. (G) mRNA expression of novel cell surface genes in osteosarcoma and normal human organs based on the TCGA and GTEx databases.

To comprehensively reveal the transcriptomic characteristics of osteosarcoma, we performed Oxford Nanopore Technologies (ONTs) long‐read RNA‐Seq of tumor and adjacent normal tissues from 23 patients with osteosarcoma. In total, 4699 differentially expressed genes (DEGs) were identified (criteria: |fold change| ≥ 2, adjusted *p* value < 0.05) (Figure 1A and Table S1). The Kyoto Encyclopedia of Genes and Genomes (KEGG) pathway analysis of DEGs showed that the PI3K–AKT signaling pathway, involved in osteosarcoma tumorigenesis,^24^ was significantly enriched in osteosarcoma compared with adjacent normal tissues (Figure S1A and B). Among the 4699 DEGs, 2535 were upregulated in osteosarcoma and further defined as tumor‐specific genes (TSGs) (Figure 1A). The gene ontology (GO) analysis of the TSGs showed an enrichment of genes related to ATPase activity, collagen‐containing extracellular matrix, skeletal system development, and nuclear division (Figure 1B and S1C). Gene set variation analysis (GSVA) showed that these TSGs were more enriched in DNA repair and glycolysis (Figure 1C), consistent with previously reported tumor characteristics.^25^ In addition, by excluding genes that are upregulated in other cancer types compared with their corresponding normal tissues in the TCGA Pan‐Cancer Atlas dataset, we identified 609 osteosarcoma‐specifically upregulated genes (OSUGs) in the 2535 TSGs (Figure S1D and Table S2). KEGG pathway analysis showed that OSUGs were significantly enriched in pathways including herpes simplex virus 1 infection, protein processing in endoplasmic reticulum, and axon guidance (Figure S1D).

Alternative splicing (AS), a critical type of posttranscriptional regulation, could be optimally investigated by long‐read sequencing with full‐length transcript recovery.^26^ In total, 58,800 AS events in 3938 genes were detected in the 23 osteosarcoma samples and 13 adjacent normal tissues (Figure S2A and Table S3). The average number of AS events per sample was significantly higher in osteosarcoma compared with normal tissues (Figure S2B), suggesting that AS‐related variations were involved in the tumor development. These AS events were further grouped into five types, and average numbers of different AS types per sample, including exon skipping (ES), alternative 3′ splice sites (A3SS), alternative 5′ splice sites (A5SS), and intron retention (IR) were significantly higher in osteosarcoma compared with normal tissues (Figure S2C). No significant difference in average numbers was found in mutually exclusive exons (MEE) between groups (Figure S2C). Consistent with previously reported studies of AS in other cancers,^27^, ^28^ ES was the most frequent AS event, accounting for over 60% of events in both osteosarcoma and adjacent normal tissues (Figure S2D). Furthermore, the differentially expressed AS events between the 13 matched pairs of osteosarcoma and adjacent normal tissues (criteria: |ΔPSI (percent spliced in) | > 10%, adjusted *p* value < 0.01) were ranked in a descending order (Figure S2E and Table S4). These differential splicing events, such as ES in the *LMO7* and *SLC37A4* loci, were further defined as osteosarcoma‐specific AS events (Figures S2F and G and Table S4). More interestingly, expression of several genes with differential splicing events such as *LMO7*, *PTS*, and *RPS24* were significantly correlated with prognosis (Figures S2H and I). Of note, 0.28% of all splicing events, such as ES in *NDEL1* and *ACIN1*, exhibited osteosarcoma specificity but also patient heterogeneity (Figures S2F and S2J and K). These AS events might be potentially associated with the tumorigenesis of specific osteosarcoma patients.

### 2.2 Transcriptomic analysis revealing potential therapeutic targets of osteosarcoma

Next, to identify potential therapeutic gene targets in osteosarcoma, we compared TSGs with a series of genes according to their biological functions and cellular locations, including genes encoding kinases, epigenetic factors, transcription factors, metabolism‐related proteins, and cell surface proteins^29^, ^30^, ^31^, ^32^, ^33^ (Figure 1D and Table S5). A total of 91 tumor‐specific kinases (TS‐K), 83 epigenetic factors (TS‐EF), 192 transcription factors (TS‐TF), and 401 metabolism‐related genes (TS‐M) were identified (Figure 1D). We further compared these sets of TSGs with target genes of approved drugs in the DrugBank^34^ (Figure 1E and Table S6). Among them, 58 TS‐K, 20 TS‐EF, 9 TS‐TF, and 114 TS‐M were target genes of approved drugs in the DrugBank (Figure 1E). For instance, *AKT1*, *TOP2A*, *DHFR*, and *RARG* were target genes of capivasertib, DOX, MTX, and ethacridine lactate, respectively (Figure 1E). The expression level of overlapped target genes could be instructive in the future drug development (Figure S3A). Furthermore, a total of 566 TSGs (33 TS‐K, 63 TS‐EF, 183 TS‐TF, and 287 TS‐M) were identified beyond the reported approved‐drug target genes in the DrugBank (Figure 1E). More interestingly, the expression of several of these genes, including *CHST13*, *FKBP11*, *SGMS2*, *TRPS1*, *PRKX*, *SP7*, and *DNAJC1*, were highly associated with osteosarcoma prognosis (Figure S3B). These data provided a list of potential new target genes for novel drug development for osteosarcoma treatment.

ACT is increasingly promising for solid tumor therapy.^35^ We thus looked into genes encoding cell‐surface proteins in osteosarcoma from our sequencing data. Most gene targets commonly investigated in ACT of other solid tumors except osteosarcoma,^1^, ^36^ such as *CEACAM1*, *PSCA*, *MSLN*, *IL13RA2*, and *GPC3*, showed low or nonspecific expression in osteosarcoma compared with adjacent normal tissues (Figure 1F). Further, some gene targets already investigated in the ACT of osteosarcoma, including *B4GALNT1* (encoding beta‐1,4‐N‐acetyl‐galactosaminyltransferase 1 involved in the biosynthesis of GD2), *ERBB2*, and *EGFR*, also showed low expression in osteosarcoma compared with adjacent normal tissues (Figure 1F). In our data, we identified nine novel cell surface genes specifically and highly expressed in osteosarcoma (criteria: |fold change| ≥ 4 and average counts per million ≥ 90 in TSGs) (Figure 1F). More interestingly, *ALPL*, *UNC5B*, and *CADM1* were also highly and specifically expressed in osteosarcoma compared with most normal human organs based on the TCGA and GTEx databases (Figures 1F and G and S3C). Besides, expression of *UNC5B*, *CAM1*, *PTH1R*, and *FCGR3A* was significantly associated with survival of patients with osteosarcoma (Figure S3D). The above data suggested that these genes might be used as new targets of ACT for osteosarcoma therapy.

### 2.3 Identification of hub‐genes in osteosarcoma

**FIGURE 2:**
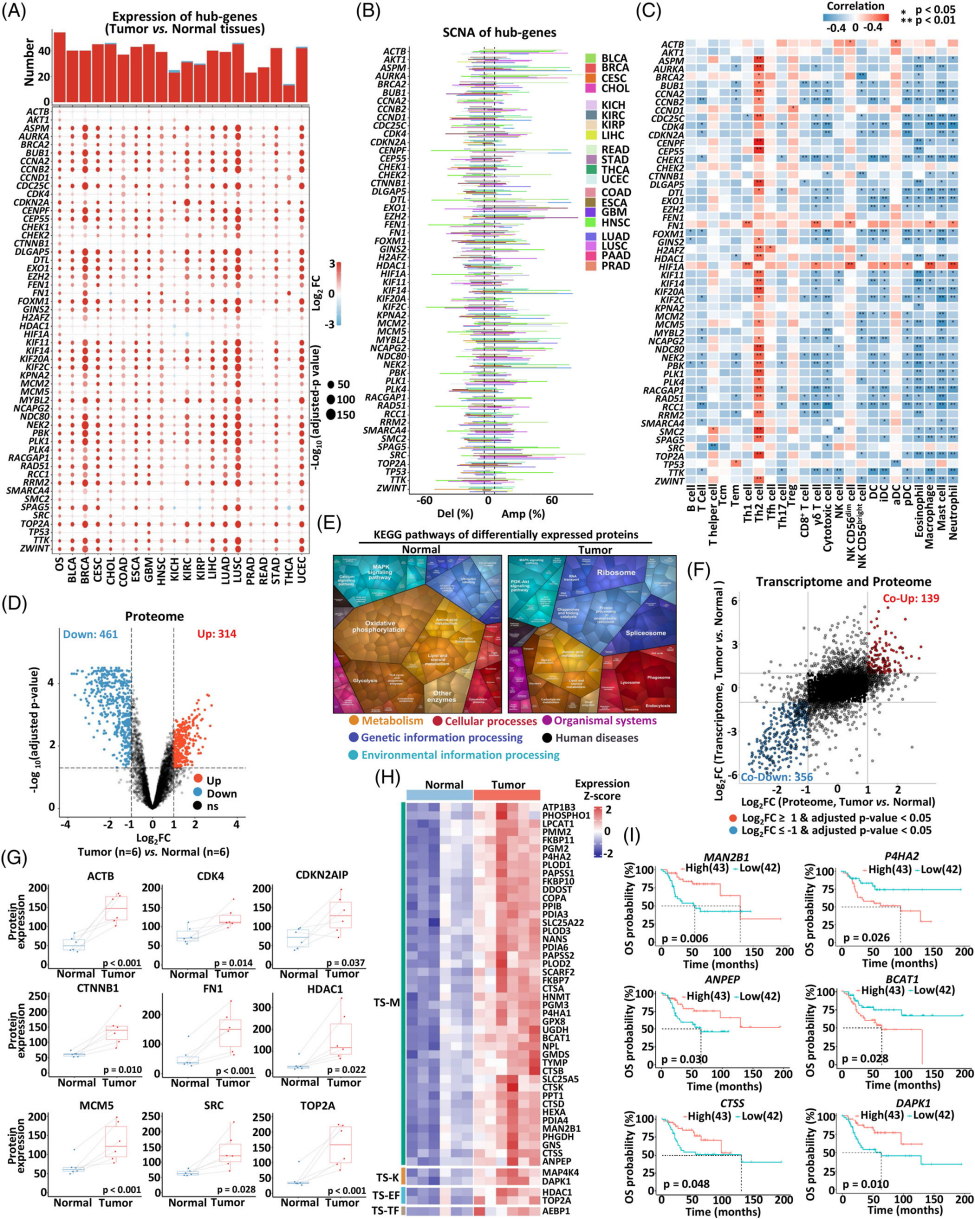
Identification of hub‐genes and proteomic profiling of osteosarcoma. (A) The number of significantly differentially expressed hub‐genes (upper panel), and the heatmap presenting the fold changes of hub‐gene expression in different tumors compared with their corresponding normal tissues (bottom panel). Abbreviations were provided in Table S17. (B) Frequency of somatic copy number alterations (SCNA) of hub‐genes at a pan‐cancer level. (C) Correlation matrix of hub‐gene expression with gene signatures of immune cells. Positive correlations are marked red and negative correlations are marked blue. (D) Volcano plot showing proteins significantly upregulated or downregulated in osteosarcoma (*n* = 6) compared with the adjacent normal tissues (*n* = 6) (criteria: |fold change| ≥ 2 and adjusted *p* value < 0.05). (E) Functional categories of differentially expressed proteins in normal tissues (left) and osteosarcoma (right) illustrated by Proteomaps. Every polygon corresponds to one KEGG pathway with the size representing the ratio. (F) Fold changes of DEGs at mRNA and protein levels. (G) Hub‐gene protein expression in osteosarcoma (*n* = 6) compared with the matched adjacent normal tissues (*n* = 6) at protein level. (H) Heatmap showing the protein expression of potential target genes in osteosarcoma (*n* = 6) and the adjacent normal tissues (*n* = 6). (I) Kaplan–Meier curve for OS probability of osteosarcoma patients in TARGET database based on the expression of indicated genes.

Hub‐genes are defined as highly connected genes in genetic interaction networks and considered to play essential roles in gene regulation and biological processes.^37^ To obtain the hub‐genes and their potential use as drug targets for osteosarcoma, a protein–protein interaction (PPI) network was constructed based on the 2535 TSGs in our transcriptome data. In total, 54 hub‐genes were identified in the shared gene list from 10 independent computational methods specific for hub‐gene identification^38^ (Figure S3E and Table S7). At the pan‐cancer level, the majority of the 54 hub‐genes were highly expressed in multiple cancer types compared with the corresponding normal tissues (criteria: |fold change| ≥ 2 and adjusted *p* value < 0.05) (Figure 2A). SCNAs are somatic changes to chromosome structure prevalent in multiple types of cancer and are the major drivers of many cellular malfunctions.^39^ Among the 54 hub‐genes, *ACTB*, *ASPM*, *AURKA*, *EXO1*, *MYBL2*, and *SRC* showed gain of copy numbers, while *TTK* and *ZWINT* showed a proclivity of copy number loss in multiple cancers (Figure 2B). The expression of most hub‐genes was negatively associated with gene signatures of several immune cell types in osteosarcoma, such as CD8^+^ T cells, cytotoxic cells, and natural killer cells (Figure 2C), suggesting that these hub‐genes might suppress immune infiltration in osteosarcoma. There were significantly increased scores of naïve CD8^+^ T cells in the group of CD8^+^ T cells with high expression of specific hub‐genes based on scRNA‐Seq data (Figure S4A). The T helper 2 (Th2) enrichment in tumor had been shown to predict worse prognosis in multiple malignancies.^40^, ^41^ Intriguingly, we observed a significant positive correlation between expression of several hub‐genes and the signatures of Th2 cells (Figure 2C), raising the possibility that high expression of these hub‐genes associated with increases of Th2 cells may contribute to worse prognosis in osteosarcoma.

### 2.4 Proteomic profiling of osteosarcoma

To gain a broad view of protein expression in osteosarcoma, quantitative TMT‐based mass spectrometry (MS) was performed with six pairs of osteosarcoma specimens and adjacent normal tissue. A total of 4974 proteins were identified (Figure 2D). Among them, 314 (6.31%) were significantly upregulated in osteosarcoma, while 461 (9.27%) were more expressed in the adjacent normal tissues (criteria: |fold change| ≥ 2 and adjusted *p* value < 0.05) (Figure 2D and Table S8). KEGG pathway analysis of the 461 proteins showed a major enrichment of oxidative phosphorylation and lipid and steroid metabolism in adjacent normal tissues (Figure 2E). In contrast, the 314 osteosarcoma‐upregulated proteins were mainly enriched in ribosome, spliceosome, phagosome, and the PI3K–AKT signaling pathway (Figure 2E). By comparing the transcriptome and the proteome data, we identified 139 upregulated genes and 356 downregulated genes at both mRNA and protein levels (Figure 2F). Fifteen of 54 hub‐genes were identified and 13 of the 15 proteins showed an upregulation in osteosarcoma compared with the adjacent normal tissues (Figures 2G and S4B). In addition, 49 out of 767 previously identified potential target genes were still identified in proteome and significantly upregulated in osteosarcoma compared with the normal adjacent tissues (Figures 1E and 2H). Among them, *MAN2B1*, *P4HA2*, *ANPEP*, *BCAT1*, *CTSS*, and *DAPK1* were significantly correlated with the prognosis of patients with osteosarcoma (Figure 2I). Further, three previously identified potential gene targets of ACT for osteosarcoma, including *CADM1*, *CDH11*, and *MMP14*, also showed high protein expression in osteosarcoma compared with normal adjacent tissues (Figures 1F and S4C). Our proteomic data further supported the clinical significance of the potential target genes identified by our transcriptome data.

### 2.5 Molecular regulation of NACT in osteosarcoma

**FIGURE 3:**
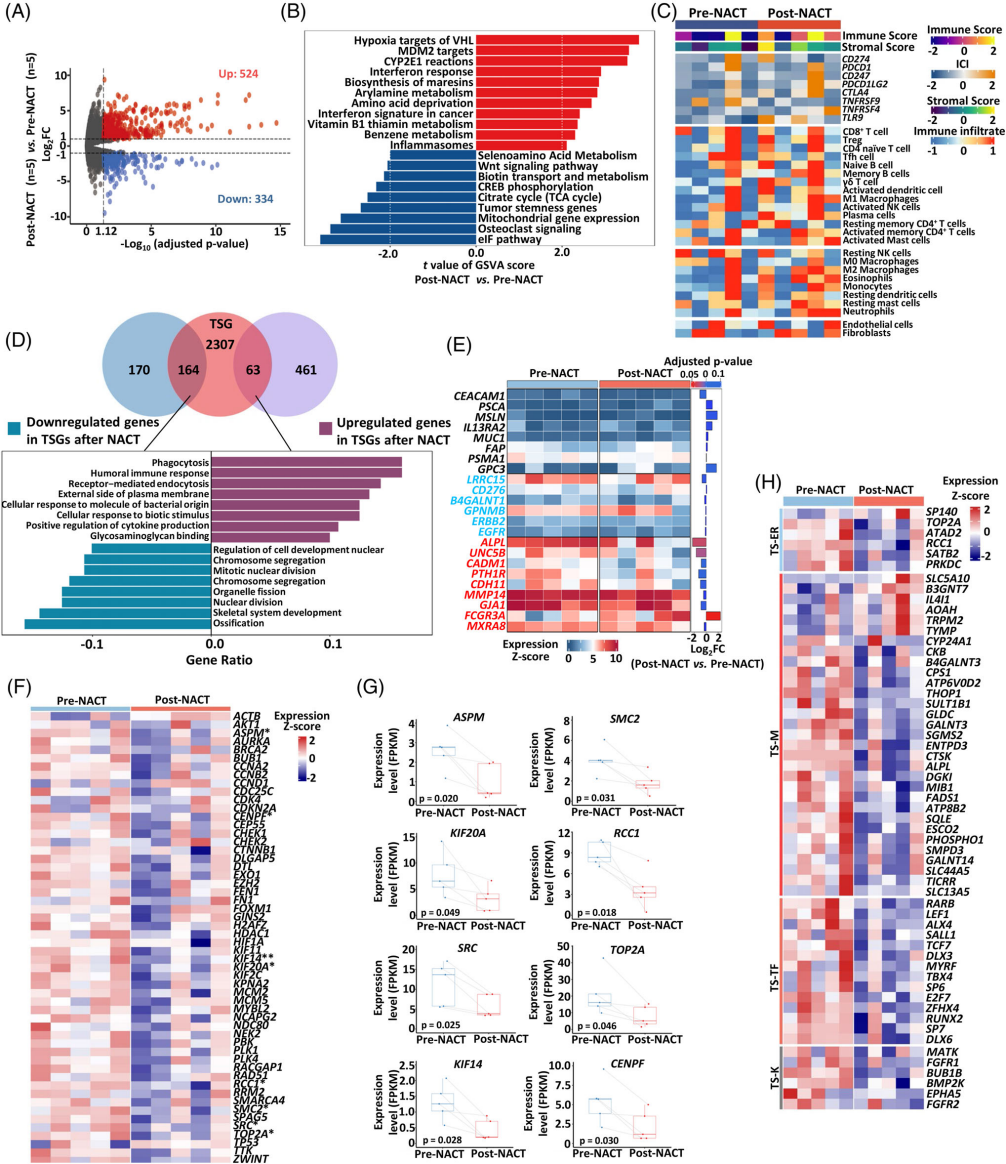
Molecular regulation of neoadjuvant chemotherapy in osteosarcoma. (A) Volcano plot of gene expression in pre‐ (*n* = 5) and post–neoadjuvant chemotherapy (NACT) osteosarcoma samples (*n* = 5) by next‐generation sequencing (NGS). (B) GSVA scores of metabolism‐related pathways between pre‐ (*n* = 5) and post‐NACT osteosarcoma samples (*n* = 5). (C) Heatmap illustrating the immune profile in pre‐ (*n* = 5) and post‐NACT osteosarcoma samples (*n* = 5). (D) GO analysis of the 63 upregulated TSGs and 164 downregulated TSGs after NACT. (E) Heatmap of mRNA expression of cell‐surface targets for ACT in osteosarcoma before and after NACT. (F) Heatmap of mRNA expression of hub‐genes in pre‐ (*n* = 5) and post‐NACT osteosarcoma samples (*n* = 5). (G) Expression levels of significantly decreased hub‐genes in pre‐ (*n* = 5) and post‐NACT osteosarcoma samples (*n* = 5). (H) Heatmap of potential therapeutic gene targets that were significantly differentially expressed between post‐ (*n* = 5) and pre‐NACT osteosarcoma samples (*n* = 5) (criteria: |fold change| ≥ 2 and adjusted *p* value < 0.05).

NACT has been one of the cornerstones for osteosarcoma treatment.^4^ To gain insight into the molecular responses of osteosarcoma to NACT, we performed next‐generation sequencing (NGS) on five pairs of pre‐NACT and post‐NACT tumor samples from five patients with osteosarcoma. We identified 858 DEGs (334 downregulated and 524 upregulated) in osteosarcoma tissues before and after NACT (criteria: |fold change| ≥ 2 and adjusted *p* value < 0.05) (Figure 3A and Table S9). GO analysis of the 524 upregulated genes showed an enrichment of immune‐related processes including humoral immune response, phagocytosis, and complement activation in post‐NACT osteosarcoma (Figure S5A). In contrast, the 334 downregulated genes were mainly enriched in skeletal developing processes including skeletal system morphogenesis and ossification (Figure S5A). Besides, metabolism‐related pathways including hypoxia targets of *VHL*, *CYP2E1* reactions and amino acid deprivation were also enriched after NACT (Figure 3B). Several amino acid transporters, such as *SLC44A5*, *SLC9B2*, and *SLC37A2*, were significantly decreased after NACT (Figure S5B), which might contribute to the amino acid deprivation in osteosarcoma after NACT.^42^ Of note, the citrate cycle pathway was less enriched in osteosarcoma after NACT (Figure 3B), suggesting a decrease activity of oxidative phosphorylation in tumor microenvironment (TME) after NACT.^43^

The GSVA showed an increase of IFN‐α response, IFN‐γ response, and TNF–α signaling via NF‐κB in post‐NACT osteosarcoma (Figure S5C), suggesting an activation of antitumor immune responses after NACT. To better understand the effects of NACT on the TME of osteosarcoma, we estimated the immune and stromal cell infiltration in osteosarcoma based on the ESTIMATE algorithm^44^ (Figures 3C and S5D and E). The stromal scores were not significantly changed after NACT (Figures 3C and S5D). However, the signature scores of overall immune cells and the innate and adaptive immune cells were increased after NACT (Figures 3C and S5E), suggesting an increasing of immune cells infiltration in the TME.

Among the previously identified 2535 TSGs, we found that NACT upregulated expression of 63 TSGs while downregulated 164 TSGs (Figure 3D). GO analysis of the 63 upregulated TSGs showed similar enrichment of certain gene sets, such as humoral immune response and phagocytosis (Figures S5A and 3D). Notably, NACT did not alter the expression of most cell‐surface protein coding genes of potential ACT targets we identified previously (Figures 1F and 3E), suggesting the potential value of using these genes as ACT targets in combination with NACT in osteosarcoma. Eight out of the 54 hub‐genes (*ASPM*, *CENPF*, *KIF14*, *KIF20A*, *RCC1*, *SMC2*, *SRC*, and *TOP2A*) were significantly decreased after NACT (criteria: |fold change| ≥ 2 and adjusted *p* value < 0.05) (Figures 3F and G), consistent with the central role of these hub genes in osteosarcoma. Fifty‐seven out of 767 potential therapeutic gene targets that showed previously were significantly changed after NACT (criteria: |fold change| ≥ 2, adjusted *p* value < 0.05) (Figures 3H and S5F and Table S10). Collectively, these results revealed the molecular regulation of NACT on the TME and potential therapeutic targets of osteosarcoma.

### 2.6 High‐throughput drug screen identification of potential therapeutic candidates and combination strategies for osteosarcoma

**FIGURE 4:**
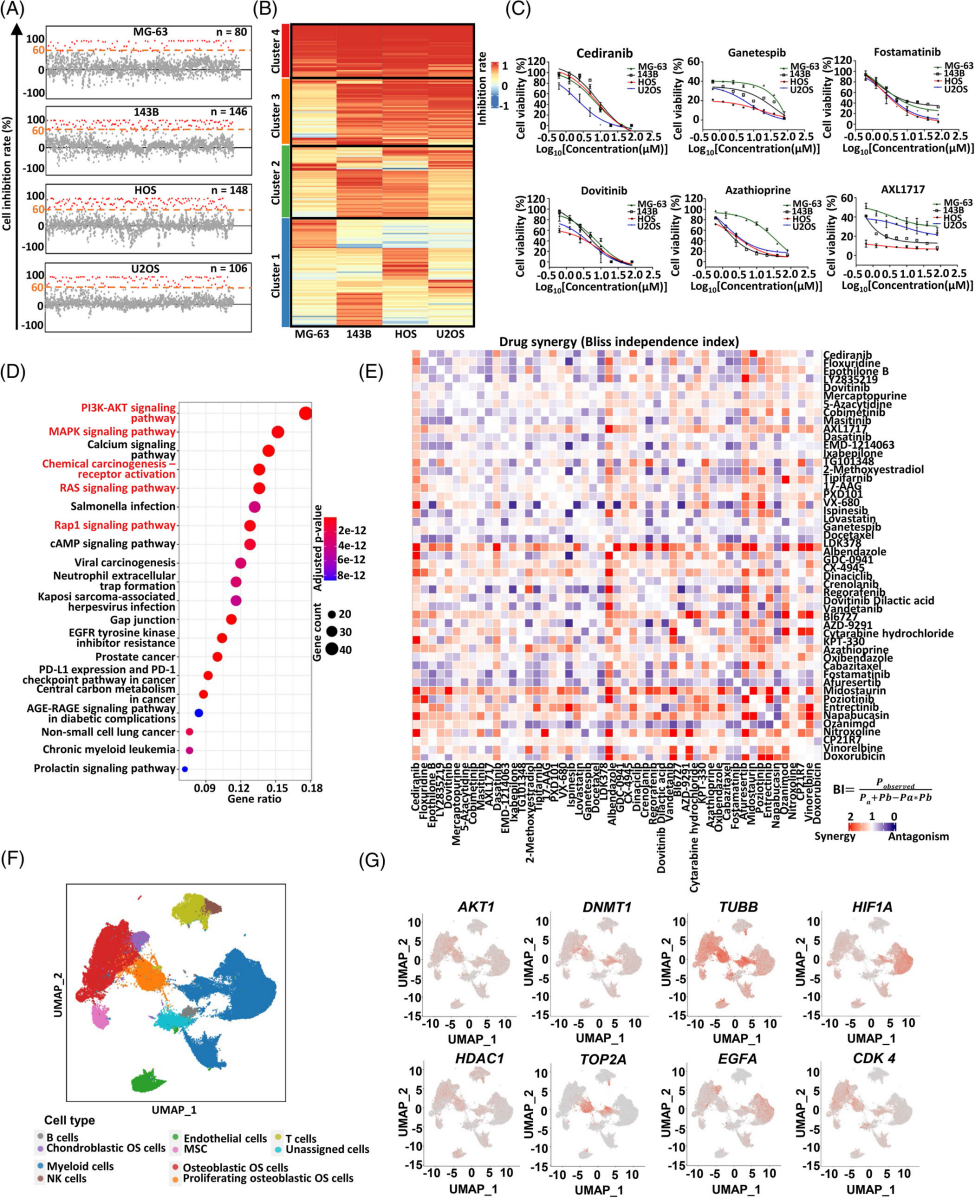
High‐throughput drug screen identification of potential therapeutic candidates and combination strategies for osteosarcoma. (A) Scatter plots of the high‐throughput screen in four human osteosarcoma cell lines (MG‐63, 143B, HOS, U2OS) with 1971 US FDA‐approved drugs (10 μM, treatment for 48 h). Drugs with significant inhibitory effect (>60%) are labeled in red. (B) Clustering of drugs with significant inhibitory effect in at least one human osteosarcoma cell lines. (C) Dose–response relationships (DRRs) of several identified drugs (*n* = 3 for each group, treatment for 48 h). (D) KEGG pathway analysis of 282 reported target genes of effective drugs. (E) Heatmap of Bliss Independences (BIs) calculated based on the screening of 50 × 50 representative effective drug combinations. The depth of red color indicates the synergistic effects of drug combinations (*n* = 3 for each drug combination). (F) Uniform manifold approximation and projection (UMAP) plot showing 10 cell types from 13 primary osteosarcoma samples (GSE162454 and GSE152048). (G) UMAPs representing the expression of target genes of effective drugs.

To identify potentially effective drugs in osteosarcoma, we first performed a high‐throughput screen with 1971 United States Food and Drug Administration (US FDA)‐approved drugs in four human osteosarcoma cell lines (MG‐63, 143B, HOS, U2OS) (Figure 4A and Table S11). At the drug concentration of 10 μM, our screen identified a total of 215 drugs with significant inhibitory effects (>60%) in at least one cell line (80 in MG‐63, 146 in 143B, 148 in HOS, and 106 in U2OS cell line) (Figure 4A and B and Table S12). The dose–response relationships (DRRs) of several identified drugs randomly selected from 215 drugs were further quantified and confirmed the reliability of our primary drug screen (Figure 4C). Based on the DrugBank and PubChem database,^34^, ^45^ we found that 179 out of 215 drugs (83.3%) had reported drug targets corresponding to a total of 282 human genes (Table S12). KEGG pathway analysis of these genes further revealed an enrichment of PI3K–AKT, chemical carcinogenesis–receptor activation, and RAS and RAP1 signaling pathways (Figure 4D), which were reported involved in tumorigenesis.^46^, ^47^ Of note, 46 out of the 282 target genes showed significantly higher expression in tumor samples compared with adjacent normal tissues (Figures S6A and B), suggesting them to be promising targets in clinical osteosarcoma treatment.

Next, we performed a combination drug screen to investigate potential synergetic effects of the above identified effective drugs. By choosing only one representative drug with the same human target genes and validating their effectiveness with DRR in all four osteosarcoma cell lines (Figure S6C), a total of 50 effective drugs were subjected to the next combination screen (Figure 4E and Table S13). In total, 1225 pairwise drug combinations were evaluated based on the Bliss Independence (BI) model^48^ (Figure 4E). As a result, a total of 236 (19.3%) drug combinations showed obvious synergistic inhibition (BI > 1.3) (Figure 4E and Table S13). Among them, notable synergistic effects were observed between the VEGFR (encoded by *KDR*) inhibitor cediranib and 30 other drugs, such as the pan‐Aurora kinase (encoded by *AURKs*) inhibitor VX‐680, ALK tyrosine kinase receptor (encoded by *ALK*) inhibitor LDK378 and cyclin‐dependent kinase 4/6 (encoded by *CDK4/6*) inhibitor LY2835219 (Figure 4E and Table S13). Besides, the multikinases inhibitor Dasatinib and ALK inhibitor LDK378 also synergistically enhanced efficacy of multiple drugs (Figure 4E and Table S13). Moreover, we noticed that DOX (targeting topoisomerase II), one of the first‐line chemotherapeutic drugs, synergistically suppressed osteosarcoma growth with pan‐HDAC (encoded by *HDACs*) inhibitor PXD101, ALK inhibitor LDK378, PRKCA (encoded by *PRKCA*) inhibitor Midostaurin, and NTRK1 (encoded by *NTRK1*) inhibitor Entrectinib (Figure 4E and Table S13).

Considering the cell‐type heterogeneity within solid tumor, we investigated expression patterns of target genes of the identified effective drugs in osteosarcoma scRNA‐Seq data from 13 patients reported previously.^19^, ^20^ (Figures S6D–G). We identified 10 major cell clusters based on reported cell markers (Figures 4F and S6H). As illustrated by feature plots, 111 of the 282 target genes of effective drugs showed significant expression in the scRNA‐Seq data (criteria: percent expression of one gene >25% in at least one cell type) (Figures 4G and S6I). Notably, 49 genes (e.g., *AKT1*, *TUBB*, *HDAC1*, and *HIF1A*) were widely expressed in most cell types (criteria: significantly expressed in at least five cell types), while nine genes (*TOP2A*, *AURKA*, *AURKB*, *CDK1*, *PLK1*, *TYMS*, *DHFR*, *GMNN*, and *RRM1*) were more specifically detected in osteosarcoma cells, especially in proliferating osteoblastic osteosarcoma cells (Figures 4G and S6I–O). The distinct expression patterns of target genes provided valuable information to guide the usage of potential drugs for osteosarcoma.

### 2.7 HDAC inhibitors synergized with DOX to suppress osteosarcoma

**FIGURE 5:**
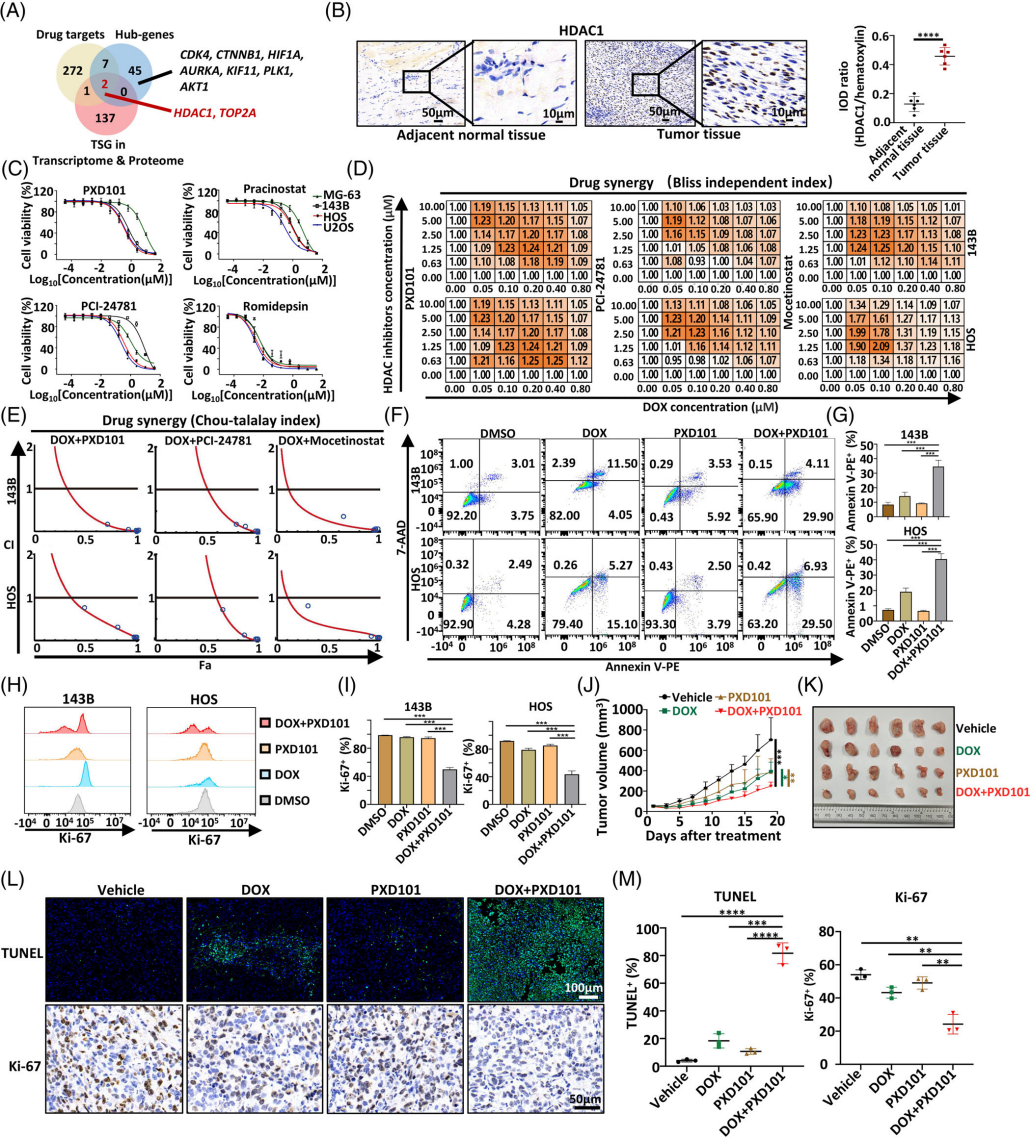
HDAC inhibitors synergized with DOX to suppress osteosarcoma. (A) Venn diagram showing overlapping genes from previously identified 139 TSGs by transcriptomic and proteomic analysis, the 54 hub‐genes and the 282 target genes of effective drugs in osteosarcoma. (B) Immunohistochemistry of images of HDAC1 staining in tumors and adjacent normal tissues. Representative images from samples of six patients. Quantification of expression level was assessed by integral optical density (IOD) ratio (the sum IOD of HDAC1 divided by sum IOD of hematoxylin staining). (C) DRRs of four HDAC inhibitors in osteosarcoma cell lines (*n* = 3 for each indicated concentration, treatment for 48 h). (D) Synergistic effects of DOX with three HDAC inhibitors were measured and calculated by the Bliss Independent Index and (E) Chou‐Talalay Index in 143B and HOS cells, at the treatment of indicated drugs concentration for 48 h. fdata from one experiment. Experiments were repeated twice. (F) Representative image of apoptosis flow cytometry of 143B and HOS cells after treatment with indicated drugs or drug combinations for 24 h (PXD101 1.5 μM, DOX 0.1 μM). (G) Quantification of Annexin V‐PE positive cells (percentage) in 143B and HOS cells shown in (F) (*n* = 3 for each group). Experiments were repeated twice. (H) Representative images of Ki‐67 staining flow cytometry of 143B and HOS cells after treatment with indicated drugs or drug combinations for 24 h (PXD101 1.5 μM, DOX 0.1 μM). (I) Quantification of Ki‐67 positive cells (percentage) in 143B and HOS cells shown in (H) (*n* = 3 for each group). Compiled data from one experiment. Experiments were repeated twice. (J) Tumor growth of subcutaneous xenograft nude mice models after treatment with indicated drugs or drug combination (*n* = 6 for each group). Compiled data from one experiment. Experiments were repeated twice. (K) Tumor image of each xenograft at the end of treatment. (L) The representative images of TUNEL and Ki‐67 staining of tumor samples from the mice with indicated treatment of drugs or drug combination. (M) Quantification of the percentage of tumor cells that stained positively for TUNEL and Ki‐67 in each group (*n* = 3 for each group). Positive percentage of a section was calculated as the average positive percentage of five randomly selected high power microscopic fields. Data are shown as mean ± SD. *p < 0.05, **p < 0.01, ***p < 0.001, ****p < 0.0001.

Interestingly, only *TOP2A* and *HDAC1* were in the shared gene list of the previously identified 139 TSGs by transcriptomic and proteomic analysis, the 54 hub‐genes and the 282 target genes of effective drugs in osteosarcoma (Figure 5A). *TOP2A* is a well‐studied and widely used gene target for osteosarcoma treatment.^49^ Our result suggested the biological and therapeutic significance of *TOP2A* and *HDAC1* in osteosarcoma. High expression of *HDAC1* was confirmed at transcriptional and protein levels in osteosarcoma compared with adjacent normal tissues (Figures 5B and S7A). In addition, the expression levels of several other HDAC family members, such as *HDAC2*, *HDAC3*, *HDAC5*, and *HDAC8* were also upregulated (Figure S7A), suggesting a potentially redundant function of HDACs in osteosarcoma.^50^ Further, four tested HDAC inhibitors, including PXD101, Pracinostat, PCI‐24781, and Romidepsin, significantly inhibited the growth of all four human osteosarcoma cell lines (Figure 5C). These results indicated the potential therapeutic value of HDAC inhibitors for osteosarcoma.

We next investigated the synergistic effects of DOX with HDAC inhibitors for osteosarcoma treatment. Three HDAC inhibitors (PXD101, PCI‐24781, and Mocetinostat) were tested in combination with DOX in vitro. As indicated, DOX with each of the three tested HDAC inhibitors synergistically inhibited growth of 143B and HOS cells (Figures 5D and E). Synergistic effects of HDAC inhibitors were also explored in combination with two commonly used chemo drugs, DDP and MTX. Of these, only DDP demonstrated a significant synergy with HDAC inhibitors in the treatment of osteosarcoma (Figure S7B). Furthermore, cotreatment of DOX with either PXD101 or PCI‐24781 strikingly increased apoptosis and reduced proliferation in 143B and HOS cells (Figures 5F–I). We further assessed the synergistic effects of DOX with HDAC inhibitors in a human 143B xenograft mouse model in vivo. As shown, DOX in combination with PXD101 (chosen as a representative HDAC inhibitor here) resulted in significantly better suppression of tumor growth compared with other treatment groups (Figures 5J and K). Increased tumor apoptosis and decreased proliferation were also observed in the DOX and PXD101 combination group, as indicated by TUNEL assay and Ki‐67 staining, respectively (Figures 5L and M). Mice were tolerated to the cotreatment of DOX and PXD101, since there was no significant decrease of average body weight and no significant increase of toxicity in solid organs in the cotreated group of mice compared with other groups (Figures S7C and D). Collectively, our results showed that DOX and HDAC inhibitors synergistically inhibited osteosarcoma growth.

### 2.8 HDAC inhibitors synergize with DOX by downregulating SP1 to transcriptionally modulate TOP2A expression

**FIGURE 6:**
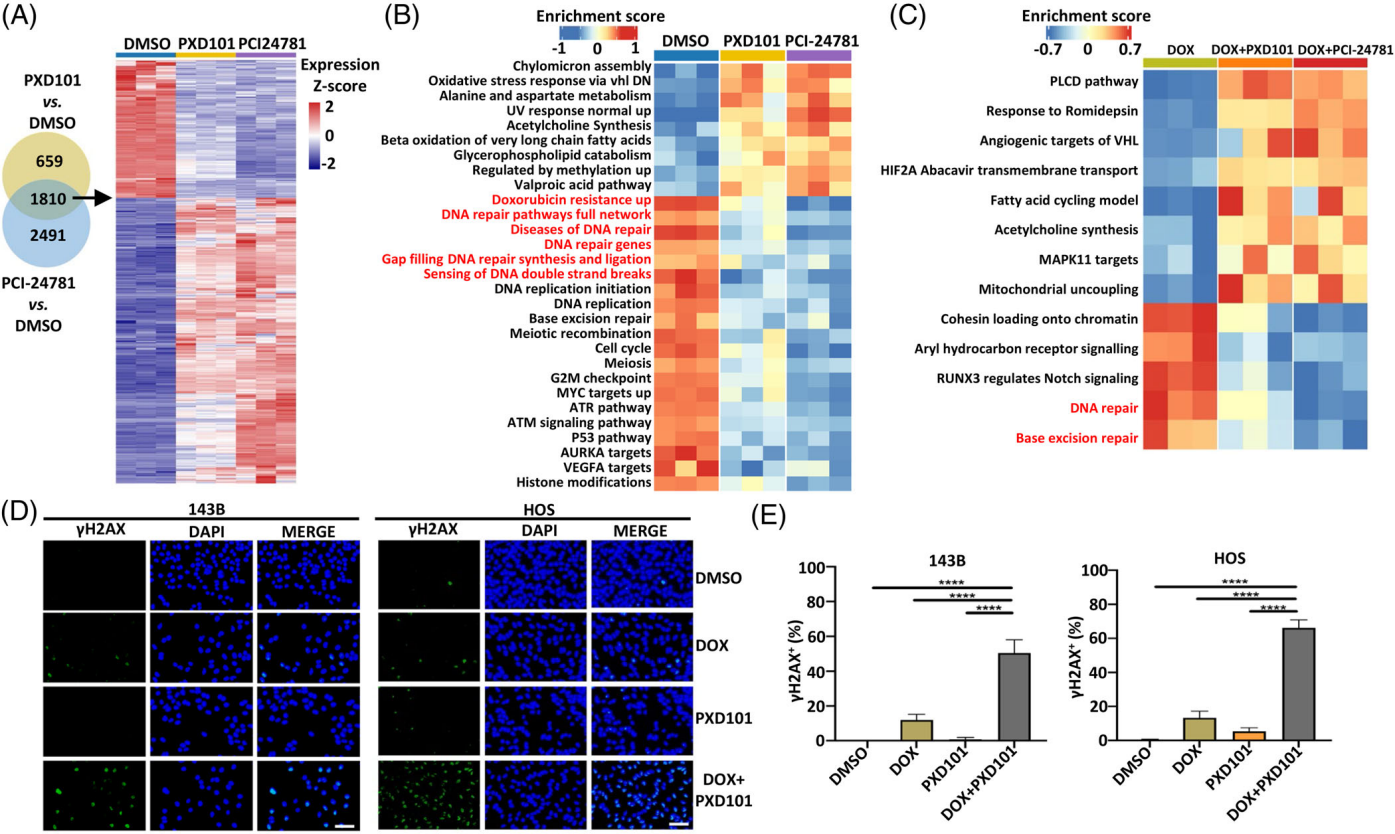
HDAC inhibitors suppress DNA repair. (A) Heatmap of DEGs identified by RNA‐seq in 143B cells after treatment with indicated drugs (*n* = 3 for each group). Cells were treated with indicated drugs for 24 h. (B) Heatmap of pathways identified by GSVA in 143B cells after treatment with indicated drugs as shown in (A) (*n* = 3 for each group). (C) Heatmap of pathways identified by GSVA in 143B cells after treatment with indicated drug or drug combinations (*n* = 3 for each group). (D) Immunofluorescence of the DNA damage marker γH2AX in 143B and HOS after 24‐h treatment with indicated drugs. Green, γH2AX staining. Blue, DAPI staining. Scale bar, 50 μm. Representative data from two independent experiments. (E) Quantification of γH2AX positive cells (percentage) in 143B cells shown in (D) (*n* = 3 for each group). Compiled data from one experiment.

To investigate the underlying synergistic mechanisms of DOX with HDAC inhibitors, we first performed transcriptomic analysis of 143B cells treated with different HDAC inhibitors including PXD101 and PCI‐24781. A total of 1810 shared DEGs were identified in PXD101‐ and PCI‐24781‐treated groups compared with controls (criteria: |fold change| ≥ 2 and adjusted *p* value < 0.05) (Figure 6A). GSVA suggested that the DOX resistance pathway was significantly downregulated in both PXD101 and PCI‐24781‐treated groups (Figure 6B), indicating that the DOX resistance could be potentially alleviated by HDAC inhibitors. In addition, the DNA repair pathways, reported to play critical role in DOX resistance,^51^ were remarkably upregulated after DOX treatment while downregulated after HDAC inhibitor treatment (Figures S8A and 6B). Furthermore, DOX in combination with HDAC inhibitors (PXD101 or PCI‐24781) markedly suppressed the DNA repair pathways compared with DOX treatment alone (Figures 6C and Figure S8B). DOX, but not PXD101, treatment alone significantly increased DNA damage in osteosarcoma cells, as indicated by significant increase expression of γH2AX (Figures 6D and E) (a biomarker of DNA damage).^52^ In contrast, DOX cotreated with PXD101 significantly increased DNA damage in osteosarcoma cells compared with DOX or PXD101 treatment alone (Figures 6D and E), suggesting that PXD101 could enhanced the DNA damage effects induced by DOX.

**FIGURE 7:**
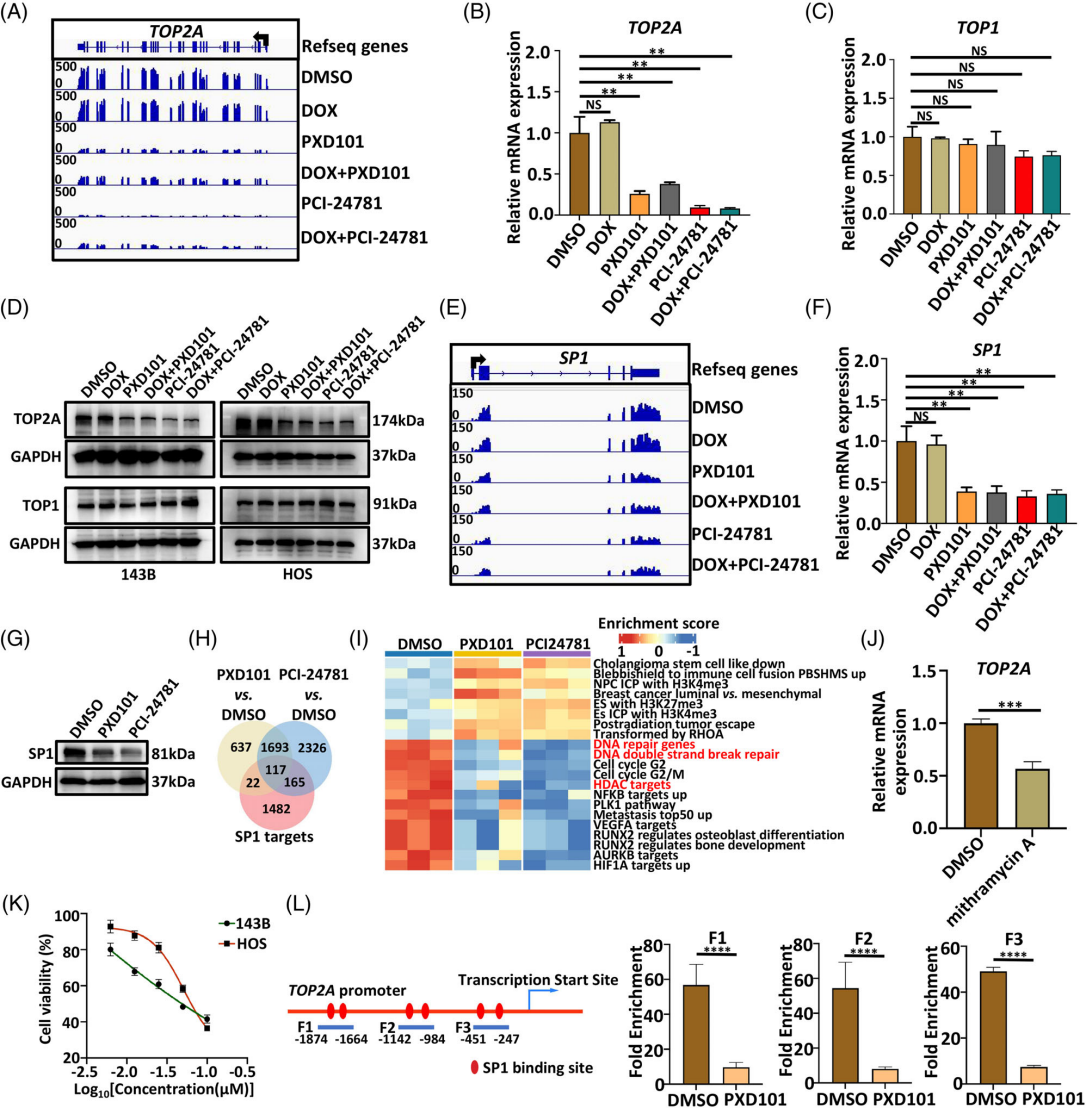
HDAC inhibitors synergize with DOX by downregulating SP1 to transcriptionally modulate TOP2A expression. (A) RNA‐seq tracks at the *TOP2A* locus in 143B cells after treatment with indicated drugs. The arrow represented the direction of transcription. (B) mRNA expression of *TOP2A* (left) and (C) *TOP1* (right) in 143B cells after treatment with indicated drugs (*n* = 3 for each group). Compiled data from one experiment. Experiments were repeated twice. (D) Protein expression of TOP2A in 143B and HOS cells after 24‐h treatment with indicated drugs by western blotting. Representative data from two independent experiments. (E) RNA‐seq tracks at the *SP1* locus in 143B cells after treatment with indicated drugs. The arrow represented the direction of transcription. (F) mRNA expression of *SP1* in 143B cells after treatment with indicated drugs (*n* = 3 for each group). Compiled data from one experiment. Experiments were repeated twice. (G) Protein expression of SP1 in 143B cells after 24‐h treatment with indicated drugs by western blotting. Representative data from two independent experiments. (H) Venn diagram comparing the DEGs identified by RNA‐seq in 143B cells after PXD101 or PCI‐24781 treatment and the *SP1* target genes identified from the Cistrome database (http://cistrome.org/db/#/). (I) Heatmap of GSVA pathways of the 117 shared genes identified in (G). (J) mRNA expression of *TOP2A* in 143B cells after indicated treatments (*n* = 3 for each group). Experiments were repeated twice. (K) Viability of 143B and HOS cells treated with mithramycin A at different concentrations (*n* = 3 for each group). Compiled data from one experiment. (L) ChIP assay of SP1 binding at promoter region of TOP2A locus in 143B cells 24 h treated with PXD101 (*n* = 3 for each group). The arrow represented the direction of transcription. Data are shown as mean ± SD. **p < 0.01, ***p < 0.001, ****p < 0.0001, NS: not significant.

Of note, transcription of *TOP2A* was significantly decreased after PXD101 or PCI‐24781 treatment alone and combined with DOX compared with controls^53^ (Figure 7A). We further confirmed that single HDAC inhibitors (PXD101 or PCI‐24781) or combined HDAC inhibitor/DOX, but not DOX treatment alone, significantly reduced the expression of *TOP2A*, but not *TOP1*, at both transcriptional and protein levels (Figures 7B–D). A previous study reported that HDAC inhibitors could directly suppress the expression of SP1, a zinc finger family transcription factor.^54^ We found that *SP1* was significantly downregulated at both mRNA and protein levels in osteosarcoma cells treated with HDAC inhibitors (Figures 7E–G). Among the reported SP1 target genes from the Cistrome database,^55^ 117 (6.6%) of 1786 genes were significantly differentially expressed both in PXD101 and PCI‐24781‐treated groups compared with controls (Figure 7H). These genes were less enriched in DNA repair pathways in PXD101 and PCI‐24781‐treated groups compared with control groups (Figure 7I). Interestingly, *TOP2A* was also among the 117 SP1 target genes (Figure 7I), suggesting that SP1 may directly regulate *TOP2A* expression. As expected, inhibition of SP1 by mithramycin A (a selective SP1 inhibitor) significantly inhibited the expression of *TOP2A* in 143B cells (Figure 7J). In addition, inhibition of SP1 by mithramycin A also resulted in a dose‐dependent suppression of 143B and HOS cell growth (Figure 7K). These results suggested that HDAC inhibitors synergize with DOX by downregulating SP1 to transcriptionally modulate *TOP2A* expression. Indeed, the chromatin immunoprecipitation (ChIP) assay analysis showed that SP1 directly bound to the promotor regions of *TOP2A*, and HDAC inhibitor PXD101 treatment significantly decreased the SP1 binding to promotor regions of *TOP2A* (Figure 7L). Collectively, our results suggested the synergistic mechanisms between DOX and HDAC inhibitors that inhibition of HDACs suppressed *TOP2A* transcription through downregulation of SP1.

## 3 DISCUSSION

It remains challenging to prolong survival or provide a potential cure for patients with osteosarcoma because of its complex and heterogeneous nature. The latest major advances in therapy against osteosarcoma were made over 30 years ago by combining DOX, DDP, MTX, and/or ifosfamide (IFO) in NACT.^4^ Novel effective therapeutic approaches for osteosarcoma are therefore urgently needed. Several studies have investigated new therapeutics such as the targeted therapy, immunotherapy, and molecularly informed precision medicine.^4^ Recently, multiomic analysis has become a promising method in revealing tumor biology and potential therapeutic targets, especially in tumors with low incidence.^56^ However, there is a paucity of comprehensive multiomic studies incorporating investigated potential targets for osteosarcoma. In the present study, we integrated the bulk and single‐cell transcriptome, proteome, and a high‐throughput drug screen to identify potential target genes and synergetic drug combinations, which helped understand more about osteosarcoma biology and further optimize treatment.

AS is a critical mechanism to increase gene complexity and plays a key role in numerous biological processes.^57^ In the present study, we identified differential AS events between osteosarcoma and adjacent normal tissues. Many of them exhibited heterogeneity between different patients. More interestingly, expression of several genes with these AS events were significantly associated with prognosis of osteosarcoma patients. These osteosarcoma‐specific or patient‐heterogenic splicing isoforms might be potential targets for osteosarcoma therapy. As the main focus was primarily on the DEGs based on expression level, the comprehensive information of AS would serve as a resource for researchers. Future investigations are required to determine the biological roles of these AS isoforms in osteosarcoma. Additionally, we found that several genes encoding cell surface proteins were more highly and specifically expressed in osteosarcoma compared with most normal human organs at transcription level. These genes could be potentially used as gene targets of ACT for osteosarcoma. However, the protein expression of these genes in osteosarcoma and in different human tissue, as well as the real effectiveness and safety of using these genes as targets of ACT need to be carefully determined.

Notably, we found that the expression of most identified hub‐genes in osteosarcoma were negatively associated with gene signatures of several immune cell types while positively associated with the gene signature of Th2 cells. As osteosarcoma is characterized by poor immune infiltration and immunosuppressive TME,^19^ these hub‐genes may be potential predictive markers for the immune situation in the TME of osteosarcoma. Our results suggested that targeting the hub‐genes may also improve immune infiltration in osteosarcoma. Indeed, NACT, which including the DOX targeting TOP2A (one of the 54 hub‐genes), significantly increased the signature scores of overall immune cells and innate and adaptive immune cells. In addition, as high infiltration of Th2 has been linked to multiple tumor progression and metastasis through induction of cytokine release and T cell anergy,^58^ our results suggested that targeting Th2 cell responses might also benefit osteosarcoma treatment; this requires further investigation.

Combinatorial drug therapy is a core strategy for osteosarcoma treatment.^4^ However, combination chemotherapy protocols have remained unchanged over the past 30 years. Recent advances in phenotypic screening could provide effective compounds for diseases without prior knowledge of treatable targets.^59^ For instance, Gu et al.^60^ conducted a high‐throughput drug screen of cells derived from 56 patients with head and neck squamous cell carcinoma (HNSCC) using 2248 compounds. Multiple drugs were identified and could be repurposed for different HNSCC subtypes. Thus, we performed a high‐throughput drug screen using 1971 US FDA‐approved compounds in four osteosarcoma cell lines and identified hundreds of potentially effective drugs for osteosarcoma. Some of these drugs were in clinical trials for osteosarcoma treatment, such as docetaxel (NCT03598595) and topotecan (NCT04661852). However, most drugs were still in preclinical phases for osteosarcoma. The integrative approach based on multiomics allowed further understanding of the target genes and corresponding signaling pathways of effective drugs. Distinct expression patterns of target genes reflected the different mechanisms of drug action. Notably, most effective drugs exerted effects by targeting the PI3K–AKT and RAS signaling pathways, which could shed light on drug application strategies.^24^

Synergistic drug combinations are promising in cancer treatment, as they can overcome compensatory mechanisms and/or increase individual drug effectiveness.^61^ In a study by Jaaks et al.,^62^ 2025 clinically relevant two‐drug combinations in 125 human cell lines using high‐throughput methods, and a landscape of drug combinations was established for tumor therapy. Similarly, we conducted a combinatorial drug screen for osteosarcoma using targeted drugs identified from our single agent screen. A total of 236 synergistic combinations with significant inhibitory effects were noticed, and these findings could provide potential strategies for future clinical trials. Furthermore, by integrating the expression patterns of target genes of synergistic drug pairs with the osteosarcoma single‐cell transcriptome, we found that cell types showed differential expression of synergistic targets. For example, *AURKA* was specifically expressed in proliferating osteoblastic osteosarcoma cells, while *CDK4* was widely expressed in all subtypes of osteosarcoma cells. *AURKA* and *CDK4* have been reported as important molecular targets mediating the cell cycle.^1^ The distinct expression patterns of target genes reflected the differences in cell types targeted by drugs. Thus, our data provide an integral understanding of combination strategies considering the effectiveness of drugs and the expression patterns of target genes.

Our multiomic data further indicated that *TOP2A* and *HDAC1* played critical roles in osteosarcoma through different analysis. The topoisomerase IIα, encoded by *TOP2A*, is responsible for topological structure transformation during gene expression and could also regulate the DNA repair.^53^ The topoisomerase IIα is the main target of DOX. HDACs could modulate the function of other proteins by deacetylating its ε‐amino lysines.^63^ HDACs are reported as promising targets for cancer therapy, and their inhibitors have been evaluated in clinical trials.^64^ Here, we further demonstrated that DOX and HDAC inhibitors synergistically suppressed osteosarcoma growth. The combination of TOP2A and HDAC inhibitors has been assessed in clinical trials for soft‐tissue sarcoma and T‐cell lymphoma, but not in osteosarcoma (NCT00878800, NCT01902225), and showed good efficacy and tolerance.^65^, ^66^ The efficacy of this combination in osteosarcoma was highlighted in our study and could provide a significant indication for clinical applications. Moreover, the underlying mechanisms of these synergistic effects were illustrated, as the HDAC inhibitors decreased the mRNA expression of *TOP2A* by the transcriptional regulation of *SP1*. Our study complements and extends previous studies on the combination of HDAC inhibitors and DOX in leukemia and Ewing sarcoma by providing a more comprehensive multiomics analysis specific to osteosarcoma.^67^, ^68^ Thus, in addition to direct effects targeting HDACs, indirect effects of HDAC inhibitors targeting DNA topoisomerase IIα resulted in an increase of DOX sensitivity. Whether coinhibition of one pathway or even the same protein (e.g., DNA topoisomerase IIα) will contribute to supra‐additive efficacy remains unclear,^69^ but DOX and HDAC inhibitors showed supra‐additive effects in osteosarcoma. Moreover, SP1 is a promising target for cancer treatment.^70^ Although we showed an *HDAC–SP1–TOP2A* regulation axis in osteosarcoma, other *HDAC* and/or *SP1* downstream pathways/targets beyond *TOP2A* may also contribute to the supra‐additive effect of HDAC inhibitors and DOX in osteosarcoma.

There are still several limitations in the current study. First, the long‐read RNA‐seq, TMT‐based quantitative proteome, and NGS RNA‐seq were conducted on different samples, which may have limited the integrative analysis. In order to avoid the impact of NACT on molecular characteristics of osteosarcoma, only tumor samples before NACT were collected. The needle biopsy can only provide a limited amount of tissue, which was not enough for multiple types of analyses. However, we analyze the data in a systematic, objective, and rigorous manner as previously reported,^71^ which could also generate valuable information and insights into osteosarcoma. Second, sample size of osteosarcoma before and after NACT is relatively small, which could limit the statistical power. There would be an interval of more than 2 months for patients with osteosarcoma to receive NACT, since the first biopsy performed. The long interval between biopsy and final surgery also increased the difficulty of collecting paired tumor samples. Only five pairs of eligible samples were collected from our cancer center at the end of this study. However, the relatively small sample size could also produce reliable results as reported.^72^, ^73^ In this study, we elucidated the molecular landscape of osteosarcoma before and after NACT based on these precious samples, which would improve understanding of the influence of NACT on osteosarcoma characteristics. We are actively working to collect more samples and enrich our data in future studies. Third, the analysis of AS was not well integrated into the overall analysis for identification of potential targets. But we recognized that the comprehensive information of AS in osteosarcoma will serve as a valuable resource for researchers who are interested in AS. The AS events as promising therapeutic targets represent important goals for future studies. Last, it is important to note that our findings are based on the cohort of Chinese patients, the generalizability of our results to other populations and ethnicities requires further investigation.

Overall, our work provided an integral understanding of osteosarcoma at multiomic levels and identified synergistic drug combinations with potential clinical implications. The data presented in this study could also serve as a valuable resource and greatly augment the knowledge of therapeutics for osteosarcoma. Future studies with more osteosarcoma models and mechanisms are warranted to extend the possibility for translation to clinical trials.

## 4 METHODS AND MATERIALS

### 4.1 Study population and sample collection

The study was approved by the Institutional Review Board of Second Xiangya Hospital, and all participants provided written informed consent. Ethical approval for this study was granted under the Second Xiangya Hospital Ethical Committee. Tumor and adjacent normal tissue samples were obtained during surgery or biopsy from patients with pathologically confirmed osteosarcoma. Twenty‐three osteosarcoma samples and 13 paired normal adjacent tissue samples were analyzed using ONT long‐read RNA sequencing (Table S14). An additional six patients with paired osteosarcoma and adjacent normal tissue samples were used for MS analysis. Matched osteosarcoma samples before and after NACT from another five patients were sequenced using NGS. Pre‐NACT specimens were collected by biopsy from patients without receiving any antitumor therapy prior to the biopsy. These patients were treated with the traditional first‐line NACT composed of a cocktail of four drugs including MTX, DOX, DDP, and IFO. All samples were assessed by two independent experienced pathologists. Clinical characteristics including age, gender and primary tumor site were retrieved from medical records.

### 4.2 Long‐read transcriptome sequencing

RNA samples were extracted from specimens, and cDNA libraries were constructed following the standard ONT long‐read RNA sequencing protocol for SQK‐PCS109.^74^ cDNA PCR was performed using LongAmp Taq Master Mix (New England Biolabs, Ipswich, MA, USA). The adapters needed for sequencing the DNA fragments were ligated using T4 DNA ligase (New England Biolabs). Amplified libraries were purified on Agencourt AMPure XP beads. Final library sequencing was performed using FLO‐MIN109 flowcells based on the PromethION platform at Biomarker Technology Company (Beijing, China). Sequencing reads were mapped to the reference genome using minimap2.^75^ Alignments of coverage <85% and identity <90% were filtered out. DEseq2 was used for the differential expression analysis between groups.^76^

### 4.3 Next‐generation sequencing

Total RNA of cells and pre‐ and post‐NACT osteosarcoma specimens were isolated using the RNeasy Mini Kit (Qiagen, Hilden, Germany). Library preparation was conducted as previously described.^77^ Briefly, library preparation was performed using the TruSeq RNA sample preparation kit (Illumina, San Diego, CA, USA). The final libraries were sequenced on Illumina Novaseq 6000 by Genergy Biotechnology Co. Ltd. (Shanghai, China). Raw data were handled by Skewer and data quality was checked using FastQC v0.11.2.^78^ Clean reads were aligned to the human reference genome (hg38) using STAR. Gene expression was normalized to FPKM (fragments per kilobase of exon per million mapped reads) values.

### 4.4 TMT quantitative proteomics

Protein extracts from tissue samples were prepared and disrupted by sonication. Proteins were digested with trypsin, and TMT labeling was performed following manufacturer instructions. The resulting peptides were subjected to liquid chromatography‐MS using an EASY‐nLC 1200 (Thermo Fisher Scientific, Waltham, MA, USA). Raw data were processed with Proteome Discover 2.4 (Thermo Fisher Scientific) and compared against the Uniprot database.

### 4.5 Public bulk and scRNA‐seq data preprocessing

Publicly available bulk RNA‐Seq of osteosarcoma was obtained from the Therapeutically Applicable Research to Generate Effective Treatments (TAREGT) database (https://ocg.cancer.gov/programs/target/data‐matrix). Clinical and outcome data corresponding to the patients were also retrieved from this database. Additionally, the expression data of normal human tissues were derived from GTEx database. All above public RNA‐Seq data were downloaded via the UCSC Xena browser (https://xenabrowser.net/datapages/). To maximize compatibility and minimize batch effects between databases, RNA‐Seq data were processed as previously described.^79^ The mRNA expression matrix, SCNA data, and somatic mutation data of pan‐cancers were obtained from the Pan‐cancer TCGA dataset via the UCSC Xena browser.^80^

Raw scRNA‐Seq data were downloaded from GSE152048 and GSE162454 in the Gene Expression Omnibus (GEO) database. In the GSE152048 dataset, seven patients with primary osteosarcoma treated with NACT were included; six pre‐NACT primary osteosarcoma specimens were collected from GSE162454. The two datasets were processed into a Seurat object and filtered by removing cells expressing <300 genes, >6000 genes and those with high mitochondrial content (>10%). Further downstream analysis was composed of SCTransform, dimensionality reduction, uniform manifold approximation and projection (UMAP), and clustering analysis. Major cell types were annotated based on known marker genes.^19^, ^20^ scRNA‐Seq data were visualized using the DimPlot and FeaturePlot functions.

### 4.6 AS analysis

AS events were detected using ASTALAVISTA (v4.0)^81^ and were analyzed individually and classified into five types including ES, A3SS, A5SS, IR, and MEE. The inclusion ratios of alternative exons or introns were calculated based on PSI‐Sigma.^82^ Differential AS events between 13 pairs of osteosarcoma and matched adjacent normal tissues were identified with over 10% PSI change and adjusted *p* value < 0.01. Sashimi plots were performed using rmats2sashimiplot with grouping files. To identify differential AS events between tumor and nontumor samples (osteosarcoma‐specific), we used the criteria as previously reported.^83^ The threshold of standard deviation (SD) of PSI in osteosarcoma was set as 0.15 to further screen individually different AS events in patients.

### 4.7 Identification of hub‐genes

The Search Tool for the Retrieval of Interacting Genes/Proteins (STRING) database (https://string‐db.org/) was used to analyze PPI of TSGs at the protein level. Hub‐genes were defined as the genes exhibiting a significantly higher degree of connectivity with other genes, which play key roles in the PPI network.^37^ The CytoHubba plugin of Cytoscape was applied to screen hub‐genes based on 10 independent ranking methods, including Maximal Clique Centrality, Maximum Neighborhood Component, Edge Percolated Component, Betweenness, BottleNeck, Closeness, Degree, EcCentricity, Radiality, and Stress.^38^ Top 10% of hub‐genes within each ranking method were identified, and we further identified the intersection of them to obtain the final hub‐genes list.

### 4.8 Gene enrichment analysis

Overrepresentation enrichment analysis was performed using KEGG and GO. Single‐sample gene set enrichment analysis was performed using the H (hallmark gene sets) and C2 (curated gene sets) downloaded from Molecular Signatures Database (MSigDB) (v7.5.1; https://www.gsea‐msigdb.org/gsea/msigdb). Proteomaps were plotted to illustrate the composition and abundance as previously described.^84^ Annotations were based on the KEGG database, and the size of polygons correlated with abundance. Immune cell signatures and related scoring criteria were obtained as previously described.^44^, ^85^ The immune and stromal cell infiltration in osteosarcoma was assessed based on the ESTIMATE algorithm.^44^

### 4.9 Statistical analysis

Overall survival (OS) was defined as the length of time from enrollment to death of any cause. Kaplan–Meier survival curves were analyzed using log‐rank test. Quantitative data are presented as mean ± SD unless otherwise noted. Continuous variables were compared using Student's *t*‐test, Wilcoxon rank sum test, and Kruskal–Wallis test, while unordered categorical variables were compared using Fisher Exact test. Statistical significance is noted as ^∗^ *p* < 0.05, ^∗∗^ *p* < 0.01, ^∗∗∗^ *p* < 0.001, or ^****^ *p* < 0.0001; NS, not significant. All *p* values from multiple comparisons were adjusted using the Benjamini–Hochberg method.^86^

### 4.10 Cell lines and reagents

Human osteosarcoma cells lines (143B, HOS, MG‐63, and U2OS) were obtained from the American Type Culture Collection (Manassas, VA, USA). The 143B, HOS, and MG‐63 cell lines were cultured in Dulbecco's modified Eagle medium (Hyclone, Logan, UT, USA) containing 10% fetal bovine serum (Newzerum) and 1% penicillin–streptomycin (Gibco) at 37°C and 5% CO_2_. U2OS cell line was cultured in McCoy's 5A containing 10% fetal bovine serum (Newzerum) and 1% penicillin–streptomycin (Gibco) at 37°C and 5% CO_2_. The US FDA‐approved drug library (library: L1021) and single drug agent (PXD101, PCI‐24781, mocetinostat, DOX) were purchased from Apex Biotechnology Corp (Hsinchu, Taiwan).

### 4.11 High‐throughput drug screening and CellTiter assay

The high‐throughput drug screenings were performed on the CHIWEN automation high‐throughput platform (MegaRobo Technologies, Suzhou, China). Basically, cells were seeded in 384‐well plates at 2000 cells/well for high‐throughput drug screening. The drug library containing 1971 single agents as well as DMSO controls were prepared in a specific 384‐well plate. The above compounds were added to wells 24 h after cell seeding. After 48 h, cells were lysed in the plate with an equal volume of CellTiter (Beyotime Biotechnology, Shanghai, China) and shaken vigorously for 2 min. Luminescence was read on a Spark plate reader (Tecan, Maennedorf, Switzerland) after 10 min incubation. Relative cell viability was calculated as the ratio of the luminescence signals between drug‐loading and DMSO‐loading wells. IC_50_, the drug concentration causing 50% cell viability relative to controls, was calculated using GraphPad Prism (GraphPad, San Diego, CA, USA).

### 4.12 Drug combination screening and drug–drug interactions calculating

Cells were seeded in a 384‐well plate for drug combination screening. Concentrations of each drug were determined by their DDRs to achieve an inhibition of less than 50%. Drug combinations were administered as described above. After 48‐h coincubation, cell viability was measured using CellTiter (Beyotime Biotechnology). Drug interactions in the combination screen were calculated using the BI model.^48^ Drug synergy was defined as the total response of a combination that exceeded the presumable sum response of two single drugs.^48^ If two single agents work independently, the theoretical value of combined response of two drugs can be calculated by the sum of two fractional responses minus their product (Pt = *P*_a_ + *P*_b_ − *P*_a_ *P*_b_). BI values represent the ratio of an actual measured combination response to the presumable summed responses (BI = Po/Pt). A BI < 1 indicated antagonistic effects, BI > 1 indicated synergistic effects, and BI = 1 indicated additive effects. Meanwhile, the Chou‐Talalay combinatorial index (CI) was also used to evaluate drug synergies, calculated using the following equation: CI = DA/da + DB/db, where da and db are the IC_50_ doses of the single drugs. DA and DB refer to the concentrations of each drug in the combination that reached the IC_50_ effect during combination usage.^87^

### 4.13 Flow cytometry

Cells were seeded in six‐well plates at 100,000 cells/well on the day before drug administration. After treatment for 24 h, attached tumor cells were digested and washed in PBS two to three times. Cells were then fixed and permeated using the eBioscience™ Foxp3/Transcription Factor Staining Buffer Set (Invitrogen, Waltham, MA, USA) overnight and stained with Ki‐67 antibodies (Biolegend, San Diego, CA, USA) for 30 min. In an apoptosis assay, cells were incubated with Annexin V‐PE and 7‐AAD after digestion and washing and incubated in the dark for 10 min. Finally, the stained cells were tested using Cytek NL‐CLC. Results were analyzed using Flowjo software (Flowjo, Ashland, OR, USA).

### 4.14 qRT‐PCR

Total RNA from osteosarcoma cells was isolated using FastPure Cell/Tissue Total RNA Isolation Kit (Vazyme) following the protocol. Quality and quantity of RNA samples were evaluated using NanoDrop spectrophotometer (Thermo Scientific). Next, the extracted RNA samples were converted to cDNA with the RevertAid First Strand cDNA Synthesis Kit (Invitrogen) according to the manufacturer's instruction. The quantitative real‐time polymerase chain reaction was performed in 96‐well plate with the FastStart™ universal SYBR® Green mix (ROX). The SYBR green fluorescence was then monitored using a Bio‐Rad iQ5 Real Time PCR. The primers used in this study were shown in Table S15.

### 4.15 Western blot

Cells were seeded in 10 cm dishes at 2 × 106 cells per dish 24 h before drug treatment. After 24‐h post drug treatment, cells were lysed for protein extraction. In brief, culture media was removed and cells were washed with PBS twice. RIPA lysis buffer containing protease inhibitor was used for cell lysis. The lysate was then centrifuged to remove cell debris. Protein concentration was measured by BCA Protein Quantification Kit (Vazyme) and separated by SDS‐PAGE. After electrophoresis, protein was transferred to the nitrocellulose (NC) membrane. NC membrane was then blocked with 5% defatted milk for 1 h and probed with primary antibody against TOP2A (Abcam, Cambridge, UK; ab52934, 1: 10,000 dilution), SP1 (Abcam; ab231778, 1:1000 dilution), TOP1 (Abcam; ab109374, 1:5000 dilution), or GAPDH (Abcam; ab181602, 1:10,000 dilution) for overnight at 4°C, washed with TBST, and further incubated with the horseradish peroxidase (HRP)‐loaded second antibody against Rabbit IgG H&L (Abcam; ab6721, 1:10,000 dilution) or Mouse IgG H&L (Abcam; ab6789, 1:5000 dilution). Finally, the bands were detected using a BG‐gdsAUTO 710 scanner (Baygene).

### 4.16 Immunofluorescence

Cells were seeded in 12‐well plates and incubated overnight. After 24‐h post drug treatment, cells were fixed with 4% paraformaldehyde in PBS, washed, and incubated with anti‐γH2AX primary antibodies (ab81299, 1:100 dilution; Abcam) overnight at 4°C, and incubated with secondary antibodies for 1 h at room temperature. Nuclei were stained with DAPI (5 min at room temperature). Fluorescence images were recorded using a fluorescence microscope.

### 4.17 Immunohistochemistry

Immunohistochemistry was performed as previously described.^88^ In brief, tissues were fixed in 4% formalin for 24 h and embedded in paraffin. For clinical sample, tissue sections were incubated at 4°C overnight with primary antibodies against HDAC1 (ab109411, 1:100; Abcam). After incubation, slides were washed with PBS for three times and were treated with the secondary antibody. Labeled cells were visualized using DAB+ as a chromogen. For tumor samples from animal experiments, primary antibodies against Ki‐67 (ab16667, 1:100 dilution; Abcam) were used. Relative expression levels were calculated using Image Pro Plus software (Mediacy Cybernetics, Inc., Rockville, MD, USA) as previously described.^89^ In brief, five 400‐magnification fields were randomly selected from each slice. Integrated optical density (IOD) or positive cell percentage of each field was measured, and the average IOD or positive cell percentage of the five fields was used as the expression level of the slice.

### 4.18 ChIP

The ChIP assay was performed according to protocols as previously described.^90^ Briefly, cells were formaldehyde cross‐linked and quenched with 125 mM glycine. Then, small chromatin fragments were generated by sonication (sizes from 100 to 500 bp). Fixed DNA–protein complexes were used for immunoprecipitation assay with anti‐Sp1 antibodies (5 μg for 25 μg of chromatin; ab231778; Abcam) or normal rabbit IgG antibodies. The precipitated DNA was used for PCR. The primer sequences for the promoter of *SP1* were listed in Table S16.

### 4.19 Mouse xenografts

All animal experiments were approved by the Institutional Review Board of Second Xiangya hospital (Serial number: 2022303). The male BALB/c nude mice (6 weeks old) were purchased from Vital River Laboratory (Vital River Laboratories, Beijing, China). They were housed in specific pathogen‐free conditions and fed with a standard diet and water ad libitum. The mice were injected with 5 × 10^6^ 143B cells subcutaneously at the right posterior flank. Tumor volume was measured with calipers and calculated as:

tumor volume = *π*/6 × length × width × height

After tumor establishment (the average tumor size reached about 100 mm^3^), mice were randomly divided into four treatment groups (i.p. injection every other day): vehicle, DOX (1 mg/kg/d), PXD101 (40 mg/kg/d), or their combination. Experiments ended when tumor volumes in the vehicle‐treated group reached 1000 mm^3^ to ensure minimal animal suffering.

## AUTHOR CONTRIBUTION

W. Z. and L. Q. contributed to conceptualization, data curation, formal analysis, validation, investigation, visualization, methodology, and writing—original draft and editing. ZY. L. contributed to investigation, methodology, and resources. C. W., Y. W., L. H., and ZX. L. contributed to investigation and methodology. Z. F. contributed to conceptualization, data curation, supervision, investigation, and writing—review and editing. C. T. contributed to conceptualization, resources, data curation, supervision, funding acquisition, project administration, and writing—review and editing. ZH. L. contributed to conceptualization, resources, data curation, formal analysis, supervision, funding acquisition, investigation, visualization, project administration, and writing—review and editing. All authors have read and approved the final manuscript.

## CONFLICT OF INTEREST STATEMENT

Authors Cheng‐zhi Wang, Ying Wu, Lianbin Han, Zhenxin Liu, and Zheng Fu are employees of MegaRobo Technologies Co., Ltd, but they have no potential relevant financial or nonfinancial interests to disclose. The other authors have no conflicts of interest to declare.

## ETHICS STATEMENT

The study was approved by the Institutional Review Board of the Second Xiangya Hospital, and all participants provided written informed consent. Ethical approval for this study was granted under the Second Xiangya Hospital Ethical Committee (Serial number: 2022303).

## Supporting information

Supporting information

Supporting information

## Data Availability

The Long‐read transcriptome and NGS data have been deposited in GEO, and the accession number was GSE218035 (https://www.ncbi.nlm.nih.gov/geo/query/acc.cgi?&acc=GSE218035). The MS proteomics data were deposited in the ProteomeXchange Consortium, with the accession number (PXD038452). All other data are available from the corresponding author upon reasonable request.
