# Supplementary material for: Integrated multiomic analysis and high‐throughput screening reveal potential gene targets and synergetic drug combinations for osteosarcoma therapy
Source: MedComm (2020). 2023 Jul 12;4(4):e317. doi: 10.1002/mco2.317 (PMC10338795; doi:10.1002/mco2.317)
Supplement: Supplementary file 1 — Supporting information [file MCO2-4-e317-s001.docx]

**Integrated multi-omic analysis and** **high-throughput screening reveal potential gene targets and synergetic drug combinations for osteosarcoma therapy**

**Running title:** **Multi-omics reveal osteosarcoma treatments**

Wenchao Zhang^1,2#^, Lin Qi^1,2#^, Zhongyue Liu^1,2^, Shasha He^3^, Cheng-zhi Wang^4^, Ying Wu^4^, Lianbin Han^4^, Zhenxin Liu^4^, Zheng Fu^4*^, Chao Tu^1,2*^, Zhihong Li^1,2*^

^1^Department of Orthopedics, The Second Xiangya Hospital, Central South University, Changsha, China

^2^Hunan Key Laboratory of Tumor Models and Individualized Medicine, The Second Xiangya Hospital, Changsha, China

^3^Department of Oncology, The Second Xiangya Hospital, Central South University, Changsha, China

^4^MegaRobo Technologies Co., Ltd, Suzhou, China

^#^These authors contributed equally to this work

***Corresponding authors:**

Zheng Fu, MegaRobo Technologies Co., Ltd, 277 Dongping Road, Suzhou, Jiangsu, China. E-mail: [fuzheng@megarobo.tech](mailto:fuzheng@megarobo.tech)

Chao Tu, Department of Orthopedics, The Second Xiangya Hospital, Central South University, 139 RenMin Road, Changsha, Hunan, China. Email: [tuchao@csu.edu.cn](mailto:tuchao@csu.edu.cn)

Zhihong Li, Department of Orthopedics, The Second Xiangya Hospital, Central South University, 139 RenMin Road, Changsha, Hunan, China. Email: [lizhihong@csu.edu.cn](mailto:lizhihong@csu.edu.cn)

**Supplementary Figures**

**
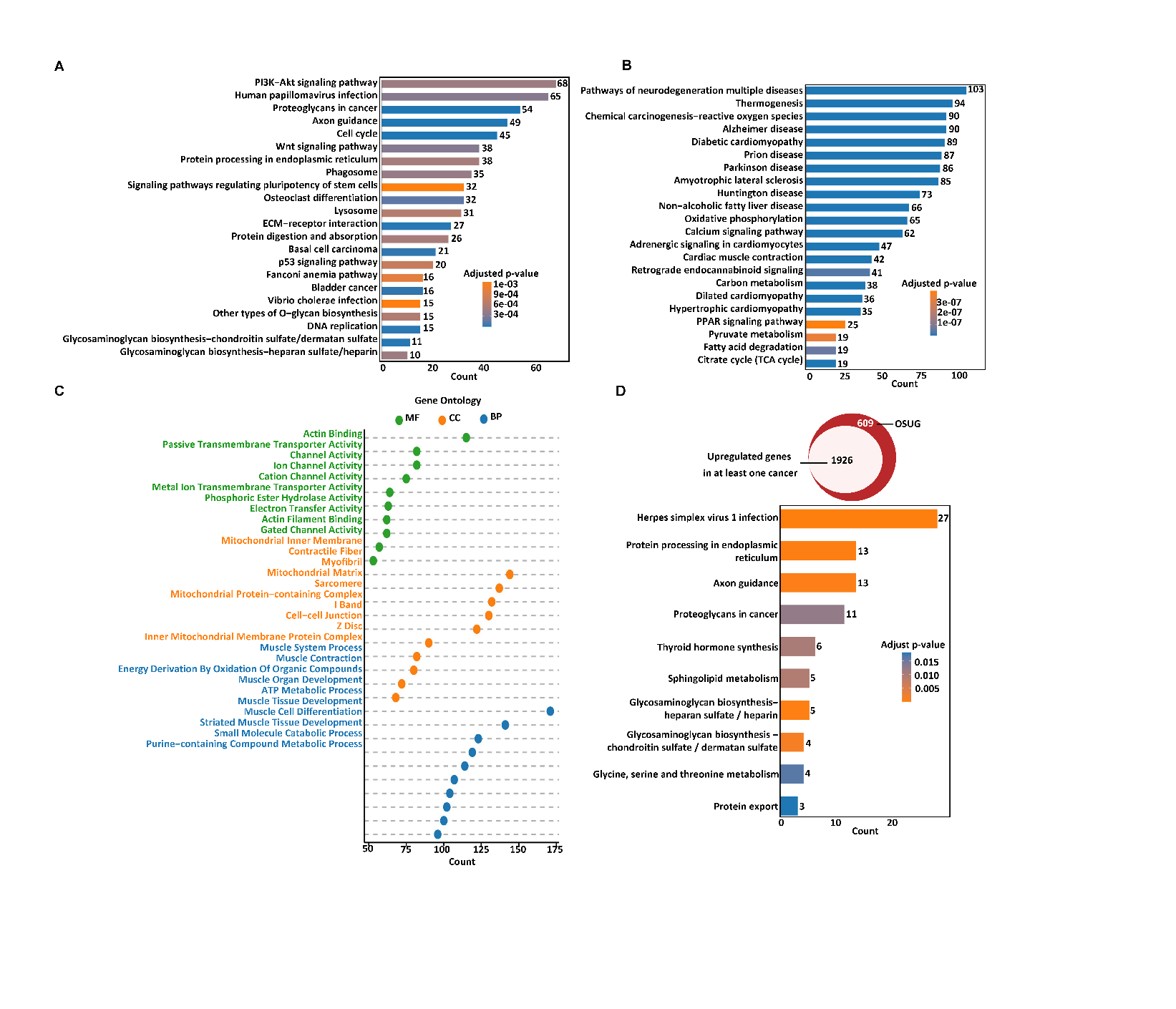
**

**FIGURE S1 Transcriptomic profiling of osteosarcoma.** KEGG pathway enrichment analysis of upregulated (A) and downregulated (B) genes between osteosarcoma (n=23) and the adjacent normal tissues (n=13). (C) GO analysis of DEGs downregulated in osteosarcoma. (D) The osteosarcoma-specifically upregulated genes (OSUGs) identified from TSGs (up) and the KEGG pathway enrichment analysis of OSUGs (down).

**
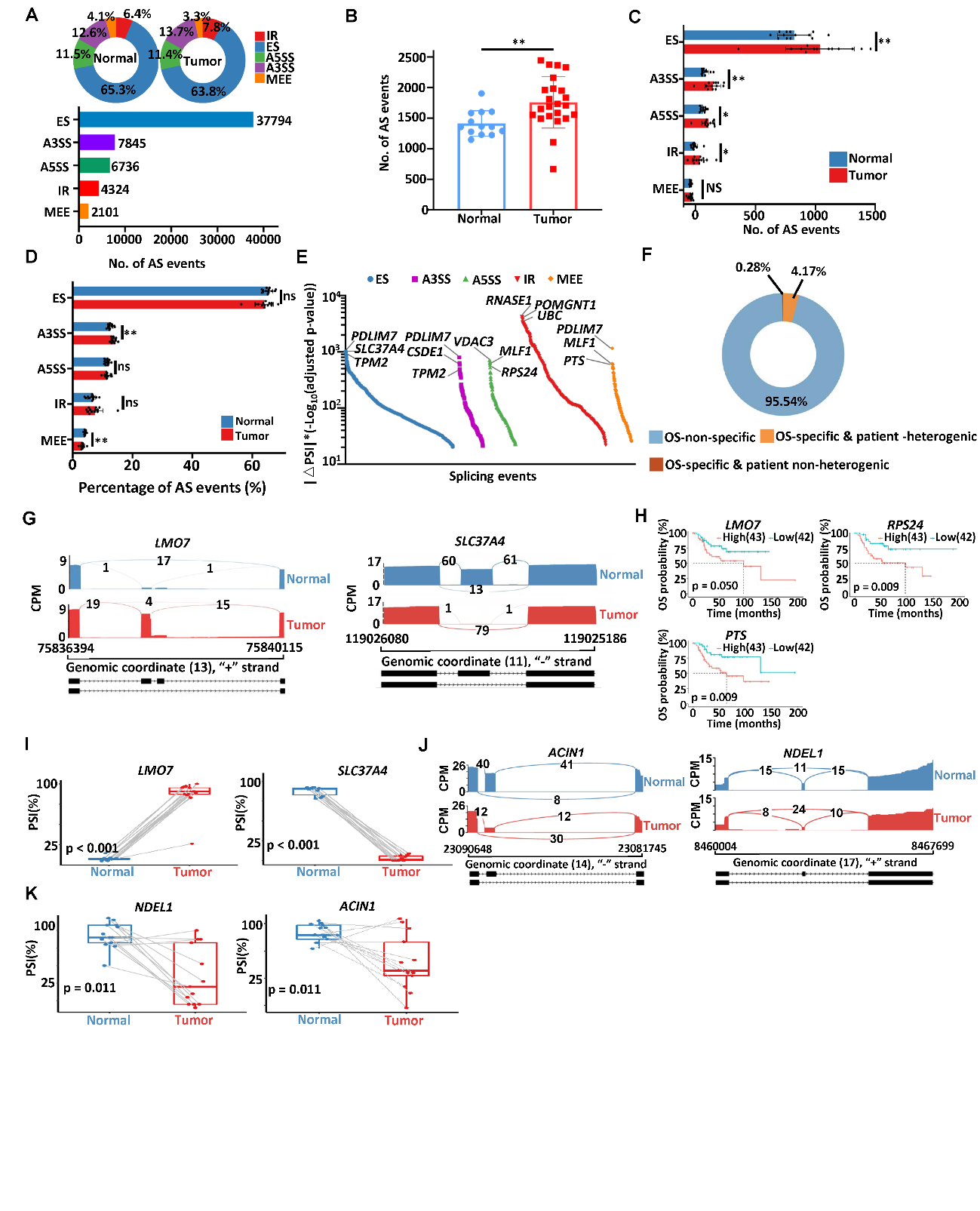
FIGURE S2 Profiling of AS in osteosarcoma**

(A) The proportion and total numbers of identified AS events in osteosarcoma (n=23) and adjacent normal tissues (n=13). (B-C) The average number of total AS events and different AS types per sample in osteosarcoma (n=23) compared to normal tissue (n=13). Exon skipping (ES), alternative 3′ splice site (A3SS), alternative 5′ splice site (A5SS), intron retention (IR) and mutually exclusive exon (MEE). (D) The average proportion of different AS types per sample in osteosarcoma (n=23) compared to adjacent normal tissue (n=13). (E) Number of differential splicing events between paired osteosarcoma (n=13) and adjacent normal tissues (n=13) (|ΔPSI (percent spliced in)| > 10%, adjusted p-value < 0.01) were ranked by|△PSI|*(-Log10(adjusted p-value)) based on the types of splicing events. (F) The proportion of OS-specific and patient-heterogenic splicing events. (G) Sashimi plots illustrating ES of LMO7 and SLC37A4 in osteosarcoma (n=13) and normal tissues (n=13). (H) Kaplan–Meier curve for OS probability of osteosarcoma patients from TARGET database based on the expression of genes with differential splicing events. (I) Differences in PSI of splicing events of LMO7 and SLC37A between paired osteosarcoma (n=13) and adjacent normal tissues (n=13). (J) Sashimi plots illustrating ES of *ACIN1* and *NDEL1* in osteosarcoma (n=13) and adjacent normal tissues (n=13). **p < 0.01, NS: not significant. (K) Differences in PSI of splicing events of NDEL1 and ACIN1 between paired osteosarcoma (n=13) and adjacent normal tissues (n=13). Data are shown as mean ± standard deviation (SD). *p < 0.05, **p < 0.01, NS: not significant.

**
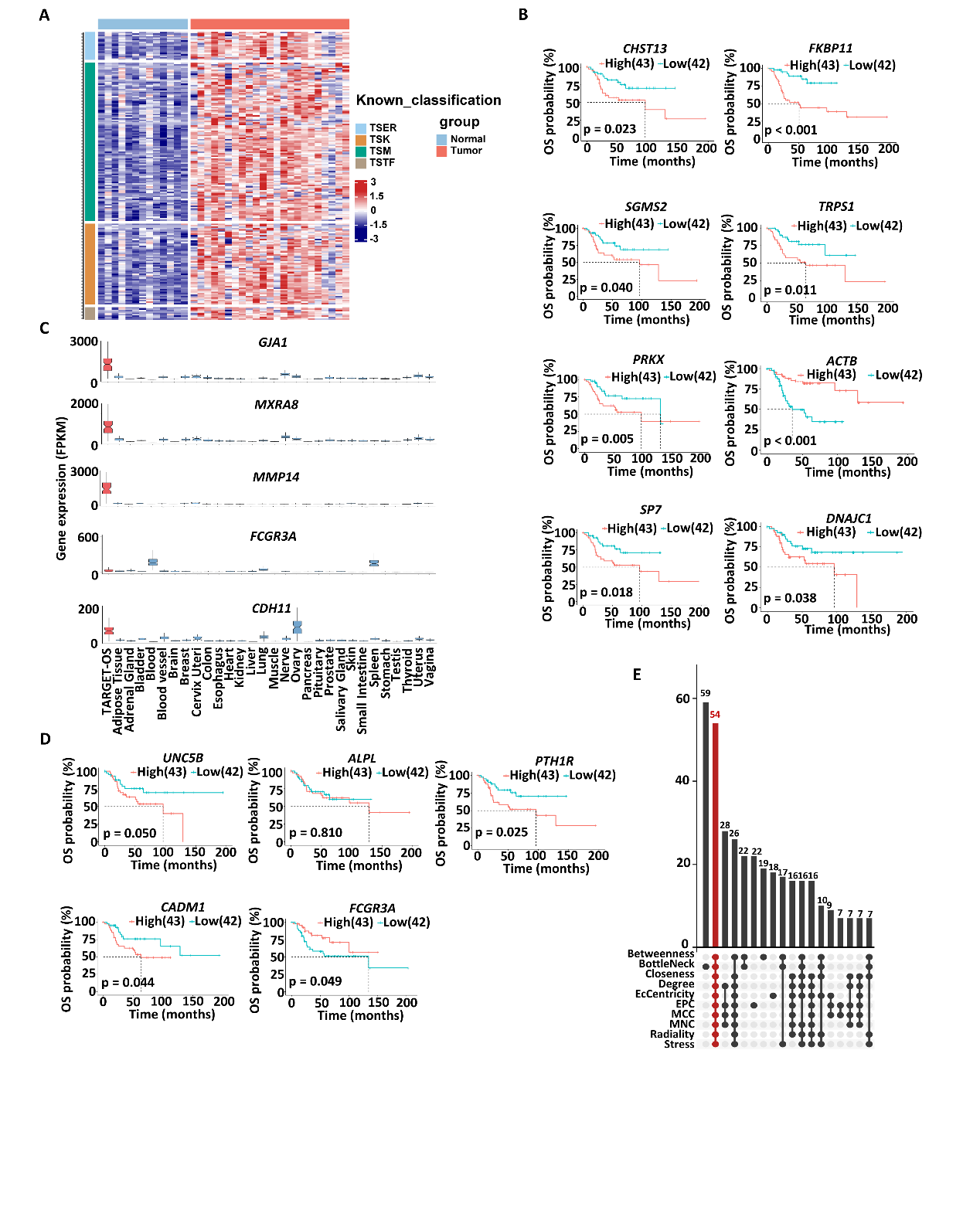
**

**FIGURE S3 Potential target genes in osteosarcoma.** (A) Heatmap of mRNA expression of potential target genes between osteosarcoma (n=23) and the adjacent normal tissues (n=13) (criteria: |fold change| ≥ 2 and adjusted p-value < 0.05). (B) Kaplan–Meier curve for OS probability of osteosarcoma patients from TARGET database based on the expression of TSGs beyond the reported target genes of the approved drugs in the Drugbank. (C) mRNA expression of novel cell surface genes in osteosarcoma and normal human organs based on the TCGA and GTEx databases. (D) Kaplan–Meier curve for OS probability of osteosarcoma patients in TARGET database based on the expression of indicated genes. (E) Identification of hub-genes with ten independent computational methods. Data are shown as mean ± standard deviation **
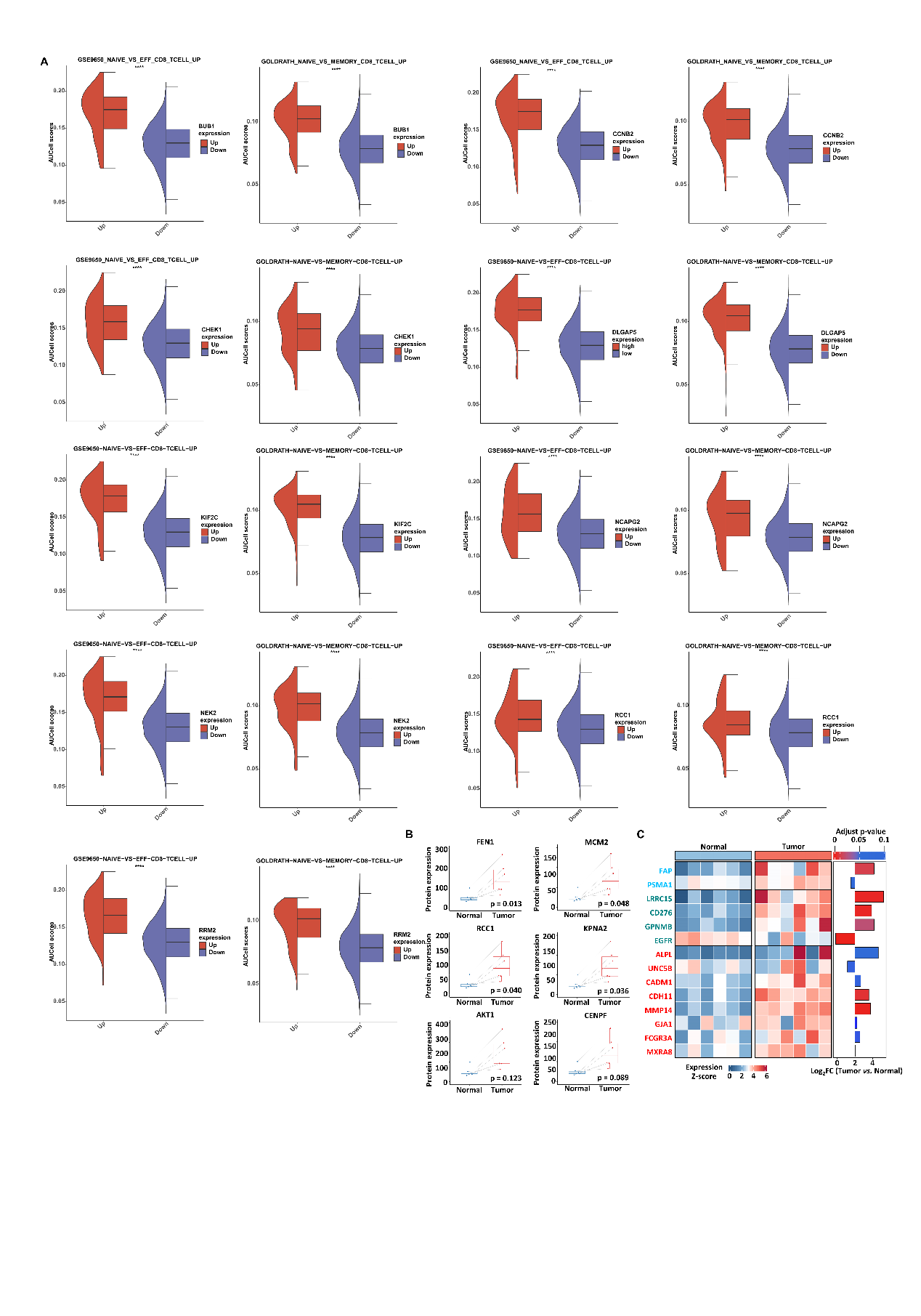
**(SD).

**FIGURE S4 Identification of hub-genes and proteomic profiling of osteosarcoma.** (A) Raincloud plots showing the enrichment scores of CD8+ T cells between groups with high and low expression of hub-genes by using AUCell. (B) Paired boxplot of the protein expression of hub-genes in osteosarcoma (n=6) compared to the matched adjacent normal tissues (n=6). (C) Heatmap of protein expression of cell-surface targets for ACT between osteosarcoma (n=6) and normal adjacent tissues (n=6).


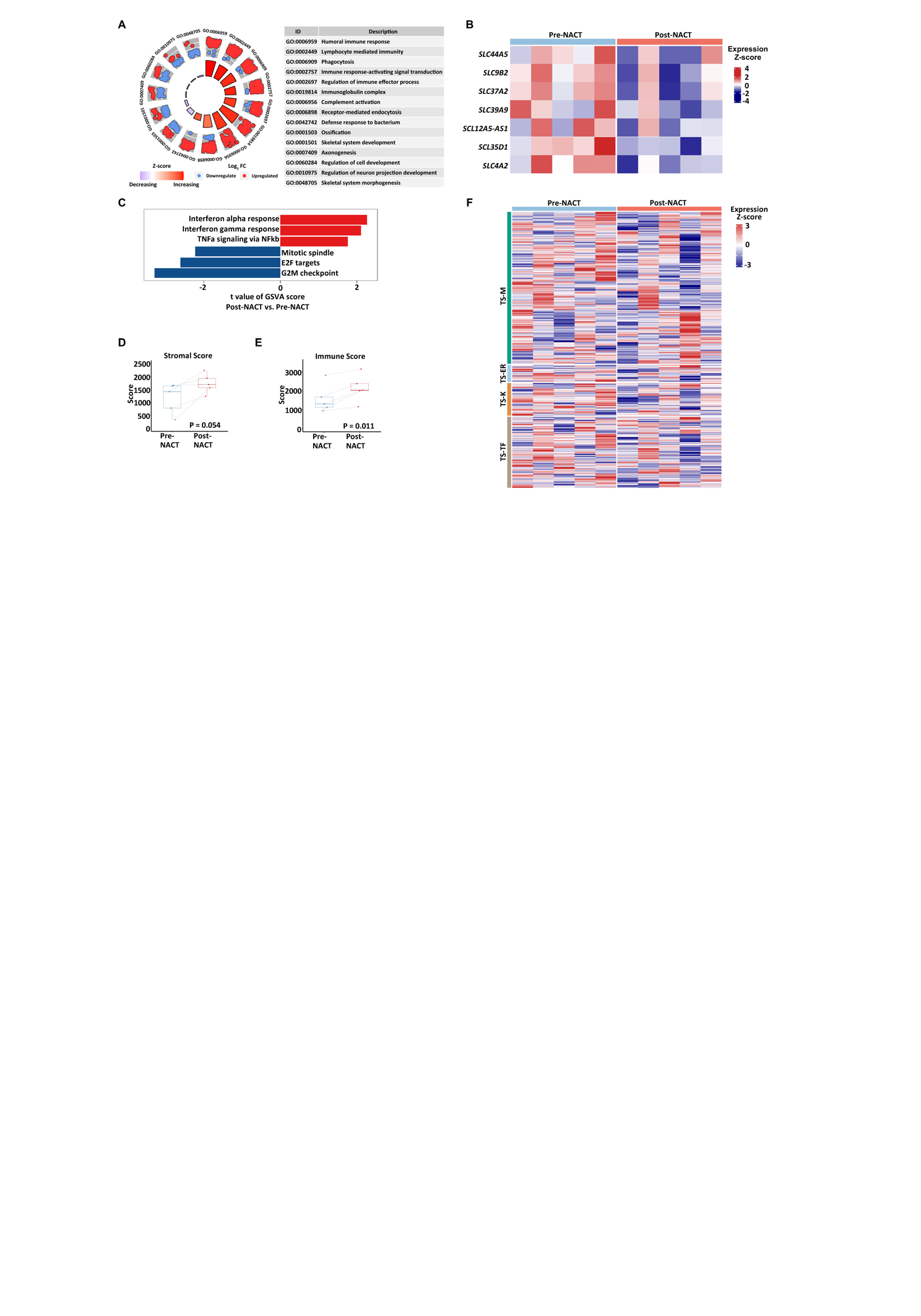


**FIGURE S5** **Molecular regulation of neoadjuvant chemotherapy in osteosarcoma.** (A) GO analysis of DEGs in pre- and post-NACT osteosarcoma samples. (B) Heatmap of mRNA expression of amino acid transporters in pre- (n=5) and post-NACT osteosarcoma samples (n=5). (C) Differences in hallmark pathway scored by GSVA between pre- (n=5) and post-NACT osteosarcoma samples (n=5). (D-E) Paired boxplot of Stromal and Immune score in pre- (n=5) and post-NACT osteosarcoma samples (n=5). (F) Heatmap of potential therapeutic gene targets that was non- differentially expressed between post- (n=5) and pre-NACT osteosarcoma samples (n=5).

**
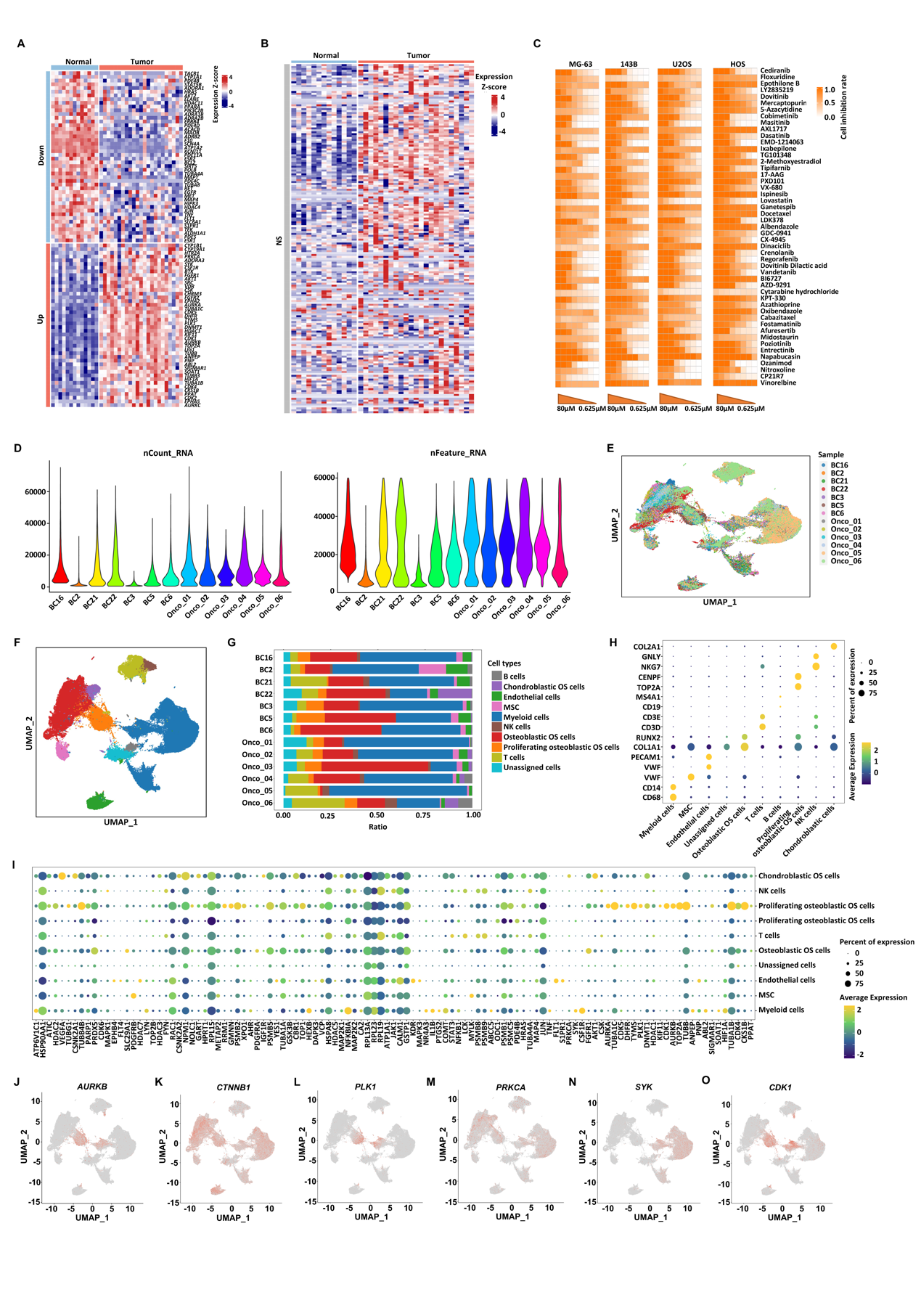
FIGURE S6** **High-throughput drug screen identification of potential therapeutic candidates and combination strategies for osteosarcoma.** (A) Heatmap showing the mRNA expression of significantly DEGs of drug targets between osteosarcoma (n=23) and the adjacent normal tissues (n=13) (criteria: |fold change| ≥ 2 and adjusted p-value < 0.05). (B) Heatmap showing the mRNA expression of non- differentially expressed drug target genes between osteosarcoma (n=23) and adjacent normal tissues (n=13). (C) DRR of representative effective drug in four human osteosarcoma cell lines (MG-63, 143B, HOS, U2OS) (n=3 for each group, treatment for 48 hours). (D) Total gene counts (nCount), number of gene types (nFeature) of the single-cell RNA-seq data from 13 primary osteosarcoma samples. (E) UMAP showing the distribution of each sample. (F-G) UMAP and barplot showing distribution of cell types in each sample. (H) Dot plot of marker genes corresponding to different cell types. (I) Dot plot of target genes significantly expressed in different cell types. (J-O) UMAPs representing the expression of target genes of effective drugs.


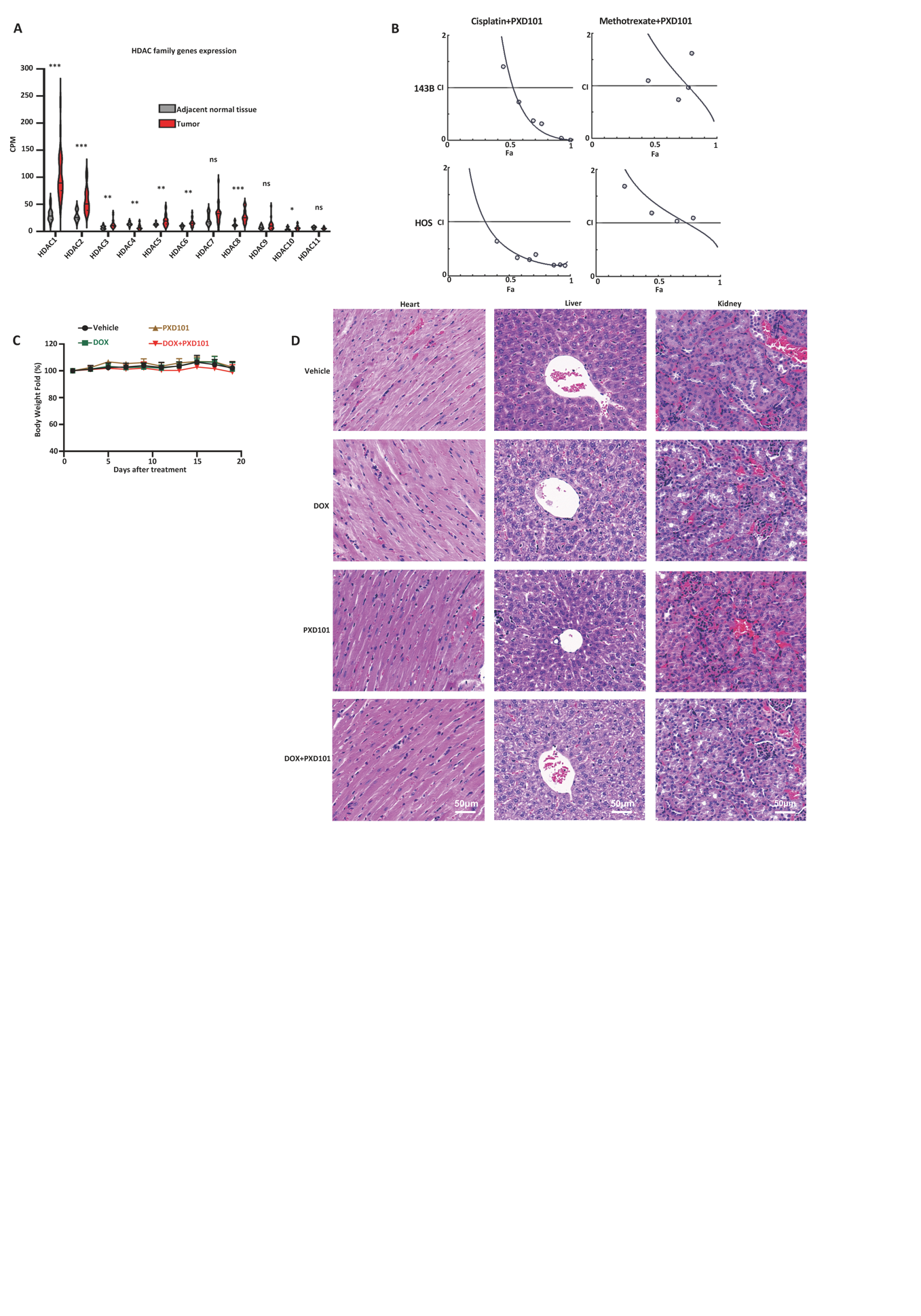


**FIGUER S7** **HDAC inhibitors synergized with DOX to suppress osteosarcoma.** (A) Violin plots showing the mRNA expression of HDAC family in osteosarcoma (n=23) and adjacent normal tissues (n=13). (B) Chou-talalay Index in 143B and HOS cells, at the treatment of indicated drugs concentration for 48 hours. Compiled data from one experiment. Experiments were repeated twice. (C) Body weight of the mice during treatment. (D) H&E staining histology of heart, liver and kidney from the mice with indicated treatment of drugs or drug combination.

**
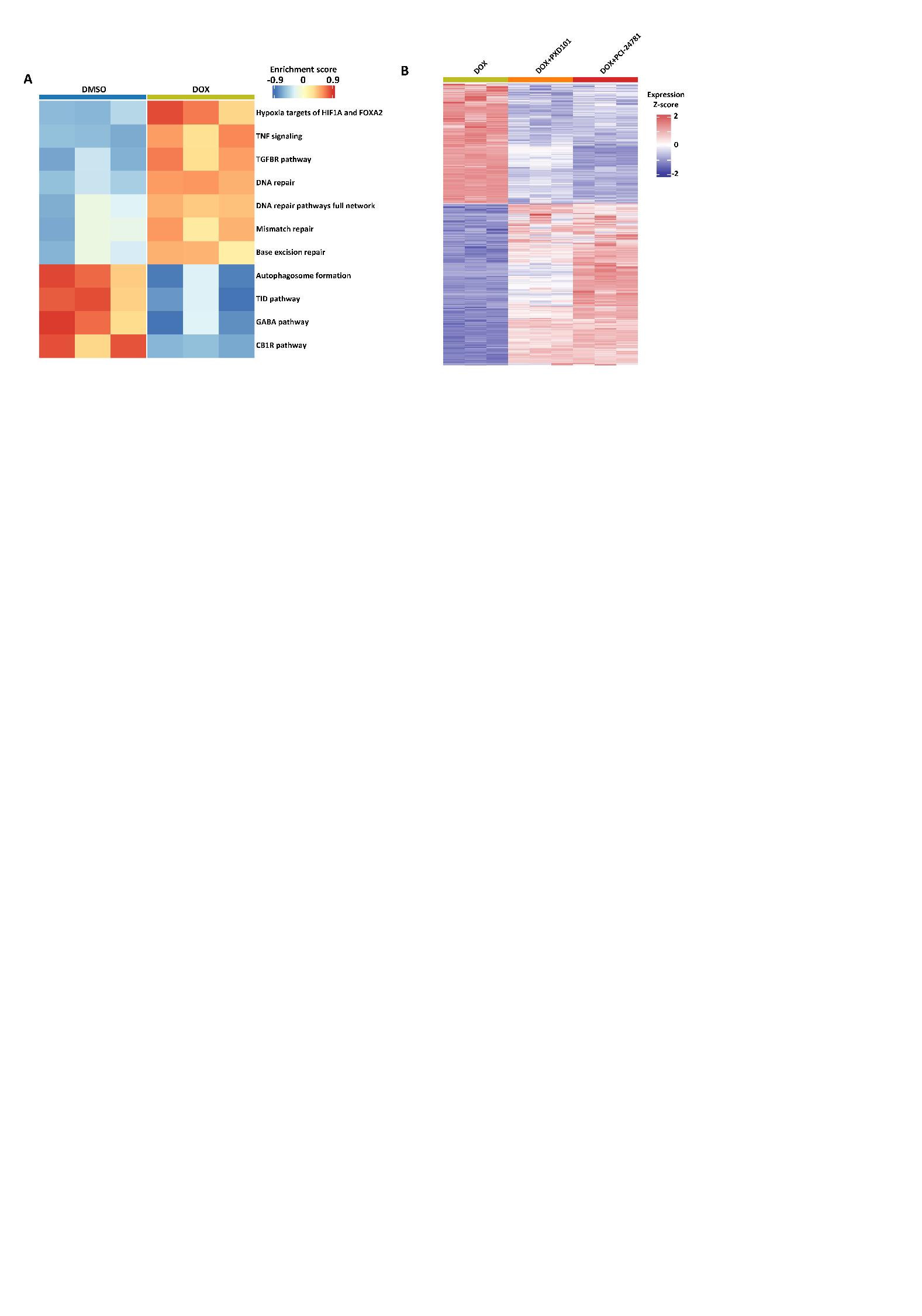
FIGURE S8** **HDAC inhibitors synergize with DOX by downregulating SP1 to transcriptionally modulate TOP2A expression.** (A) Heatmap of pathways identified by GSVA in 143B cells after treatment with DMSO or DOX (n= 3 for each group). (B) Heatmap of DEGs identified by RNA-seq in 143B cells after treatment with indicated drugs or drug combinations (n= 3 for each group).
